# Induction, regulation and roles of neural adhesion molecule L1CAM in cellular senescence

**DOI:** 10.18632/aging.101404

**Published:** 2018-03-28

**Authors:** Blanka Mrazkova, Rastislav Dzijak, Terezie Imrichova, Lenka Kyjacova, Peter Barath, Petr Dzubak, Dusan Holub, Marian Hajduch, Zuzana Nahacka, Ladislav Andera, Petr Holicek, Pavla Vasicova, Olena Sapega, Jiri Bartek, Zdenek Hodny

**Affiliations:** 1 Department of Genome Integrity, Institute of Molecular Genetics of the ASCR, Prague 14220, Czech Republic; 2 Institute of Chemistry, Slovak Academy of Sciences, Bratislava 84538, Slovakia; 3 Institute of Molecular and Translational Medicine, Palacky University, Olomouc 771 47, Czech Republic; 4 Laboratory of Molecular Therapy, Institute of Biotechnology of the ASCR, Prague 14220, Czech Republic; 5 Laboratory of Immunological and Tumour Models, Institute of Molecular Genetics of the ASCR, Prague 14220, Czech Republic; 6 Danish Cancer Society Research Center, Copenhagen DK-2100, Denmark; 7 Division of Genome Biology, Department of Medical Biochemistry and Biophysics, Karolinska Institute, Stockholm, Sweden

**Keywords:** mass spectrometry, SILAC, proteomics, MAPK pathway, aging

## Abstract

Aging involves tissue accumulation of senescent cells (SC) whose elimination through senolytic approaches may evoke organismal rejuvenation. SC also contribute to aging-associated pathologies including cancer, hence it is imperative to better identify and target SC. Here, we aimed to identify new cell-surface proteins differentially expressed on human SC. Besides previously reported proteins enriched on SC, we identified 78 proteins enriched and 73 proteins underrepresented in replicatively senescent BJ fibroblasts, including L1CAM, whose expression is normally restricted to the neural system and kidneys. L1CAM was: 1) induced in premature forms of cellular senescence triggered chemically and by gamma-radiation, but not in Ras-induced senescence; 2) induced upon inhibition of cyclin-dependent kinases by p16^INK4a^; 3) induced by TGFbeta and suppressed by RAS/MAPK(Erk) signaling (the latter explaining the lack of L1CAM induction in RAS-induced senescence); and 4) induced upon downregulation of growth-associated gene ANT2, growth in low-glucose medium or inhibition of the mevalonate pathway. These data indicate that L1CAM is controlled by a number of cell growth- and metabolism-related pathways during SC development. Functionally, SC with enhanced surface L1CAM showed increased adhesion to extracellular matrix and migrated faster. Our results provide mechanistic insights into senescence of human cells, with implications for future senolytic strategies.

## Introduction

Human population is aging rapidly, raising serious global health and societal concerns, at the same time highlighting the urgent need to better understand the process of aging at the molecular, cellular and organismal levels. Recent research has revealed aging-associated accumulation in diverse tissues of the so-called senescent cells (SC) which, along with their secreted products, appear to causally contribute to aging [1,2]. From a broader perspective, the process of senescence is a cellular stress response leading to persistent activation of cell-cycle checkpoints and long-lasting cell-cycle arrest caused predominantly by lasting DNA damage signaling. SC play important pathophysiological roles in a number of processes during ontogenetic development of the mammalian organism [3]; for instance, senescence response takes part in wound healing [4] and tissue regeneration [5]. As a cellular fate commonly triggered by DNA damage response, cellular senescence is considered as a primary tumorigenesis barrier [6–8]. DNA damage-induced cellular senescence also accompanies genotoxic cancer therapies [9,10]. Long-lasting persistence of senescent cells in tissues can also induce various pathological changes affecting organismal homeostasis (see, e.g. references [1,2]). These effects are thought to be elicited by secretion from SC of a complex mixture of factors comprising components of the extracellular matrix, its modifiers such as proteases and their regulators, reactive oxygen species [11] and particularly cytokines including morphogens from the TGFβ family, pro-inflammatory species (e.g., IL1, IL6, IL8 [12];), and chemokines (such as MCP-1 [13];). The composition of the SC secretome (denoted as senescence-associated secretory phenotype; SASP) varies according to the cell type and mechanism of senescence induction [14]. Additional factors influencing the secretory response of SC, such as interactions of SC with surrounding cells and specific tissue microenvironment, likely exist at the organismal level. This complexity and variability of SASP is the reason why the effects of SASP on the tissue microenvironment and the pathogenic role of various types of SC remain poorly understood. Nevertheless, it was plausibly demonstrated that senescent cells can affect neighboring cells by induction of oxidative stress and DNA damage resulting in secondary ('bystander') senescence [11,15,16]. This 'domino' effect might contribute to the spread of a mild but chronic inflammatory environment especially in aging tissues [17]. It should also be noted that certain chemotherapeutic drugs are able to induce cellular senescence in normal tissues within the close proximity of tumors, which can also promote local inflammation and self-sustaining genotoxic environment.

Of the various interactions of SC with their milieu, the interaction with the immune system is especially important. SC interact with the immune system in a mutual way – besides being a target of the immune system (senescence immunosurveillance [18];), SC can modulate the immune system function by either immunoactivation [18] or immunosuppression [19,20]. Intriguingly, observations that genetically engineered removal of SC from the mouse organism induced a 'rejuvenating' effect [2] and the presence of SC affected the rate of mouse aging [1] both indicate aging-promoting harmful effects of SC *in vivo*. Indeed, there is accumulating evidence that SC contribute to pathogenesis of several aging-associated degenerative diseases including atherosclerosis [21], cartilage degeneration leading to osteoarthritis [22,23], cardiac fibrosis [24], liver fibrosis [25], type 2 diabetes [26], and Alzheimer’s and Parkinson’s diseases [27,28]. In addition, elimination of senescent cells from the tumor environment is believed to improve the health condition of patients with cancer [29].

Based on the negative effects of SC in tissues, there is a prevailing view that prevention of SC accumulation or support of their elimination would be beneficial for the health span of the organism. The prerequisite for this ambition is our ability to unambiguously detect SC in tissues. However, reliable biomarkers strictly specific to SC are still missing, despite the SC undergo profound changes of their phenotype deviating extensively from their parental cells [30]. The purpose of this study was to identify candidate protein markers differentially expressed on the cell surface of proliferating versus replicatively senescent human fibroblasts using quantitative proteomic analysis. Apart from several previously reported proteins such as PVR, TIMP3, GGT2, and ADAMTS1, we identified the neural adhesion factor L1CAM as being overrepresented on the surface of the replicatively SC. Further analysis of L1CAM whose expression is physiologically restricted to neural and kidney tissues revealed that except for oncogenic Ras-induced senescence, other pro-senescence stimuli can induce L1CAM. Also intrigued by the known association of L1CAM with cancer and poor prognosis of cancer patients [31–37], we then pinpointed several cellular signaling and metabolic pathways involved in regulation to L1CAM expression, as well as functional impact of elevated L1CAM expression, using several human models of SC. Both the overall spectrum of the cell-surface proteins identified in our unbiased proteomic screen, and the insights into L1CAM regulation and potential roles in senescence are presented in this dataset.

## RESULTS

### L1CAM is enriched on the cell surface of replicatively senescent fibroblasts

**Figure 1:**
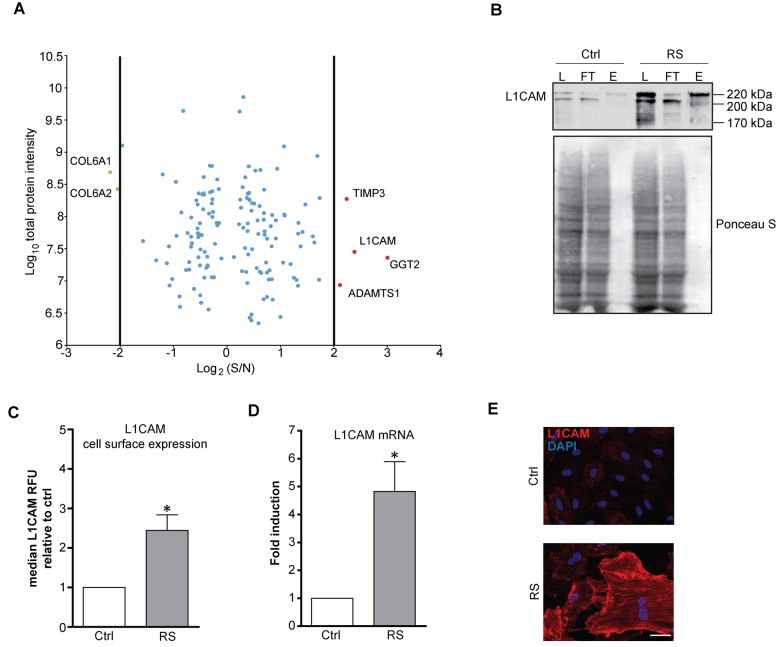
**L1CAM is enriched on the surface of replicatively senescent fibroblasts.** (**A**) Proteomic analysis of the surface of senescent cells. Down- (green dots < four fold) and up-regulated (red dots > four fold) proteins in senescent cells. (**B**) Cell surface proteins of proliferating (Ctrl) and replicatively senescent (RS) BJ fibroblasts were modified by biotin and captured on a streptavidin column (see Material and Methods for details), and fractions were validated for the presence of L1CAM by Western blotting. L – load (total protein); FT – flow through (non-biotinylated protein fraction), E – elution (biotinylated protein fraction). Ponceau S staining is shown to demonstrate protein loading. (**C**) FACS analysis of the surface level of L1CAM in BJ fibroblasts. (**D**) mRNA level of L1CAM normalized to GAPDH in replicatively senescent BJ cells. (**E**) Live cell immunofluorescence detection of L1CAM in proliferating (Ctrl) and replicatively senescent (RS) BJ (upper panel) and MRC5 (lower panel) fibroblasts. Scale bar, 50 μm. All experiments were performed in biological triplicates. For statistics, two-tailed Student’s t-test was used: p ˂ 0.05 (*); p ˂ 0.01 (**); p ˂ 0.001 (***).

In our search to identify proteins differently expressed on the plasma membrane of senescent cells, we performed quantitative proteomic analysis of cell surface proteins of proliferating (PD 28) and replicatively senescent (PD 84) BJ fibroblasts (see Supplementary Figure 1A, B for activity of senescence-associated β-galactosidase and markers of persistent DNA damage response, respectively). For enrichment of cell surface proteins we utilized biotin labeling of cell surface proteins (see Materials and Methods for details). Out of 650 quantified proteins identified by mass spectrometry, 151 proteins showed differential expression (Supplementary Table 1). Within this group we focused on the candidate proteins whose expression on the surface of senescent fibroblasts was increased more than four fold over the level in proliferating counterpart cells. In addition to several proteins already reported as overexpressed in senescent cells, such as metalloproteinase inhibitor 3 (TIMP3) [38], γ-glutamyltranspeptidase 2 (GGT2) [39] and ADAMTS1 [40], our analysis revealed some previously unrecognized proteins such as THY1, BST1, FAT1 (for full list of differentially changed proteins, see Supplementary Table 1) and neural cell adhesion molecule L1, L1CAM (Figure 1A). Thirty six most upregulated proteins were selected and analyzed for mRNA levels, of which more than dozen was significantly elevated (see Supplementary Figure 1C). The changes of total protein level of five selected proteins (fibronectin, L1CAM, collagen IV, integrin α2 and PVR) in replicative senescent BJ cells were verified by immunoblotting (see Supplementary Figure 1D).

L1CAM attracted our attention as it is expressed physiologically in neural and renal tissues and pathologically in several types of human tumors (see Discussion for further details). To validate the mass spectrometry data, we compared the L1CAM mRNA, total and surface protein expression in young/proliferating versus senescent BJ cells. As shown by immunoblotting of biotin/streptavidin immunoprecipitates, the total level of L1CAM was increased in replicatively senescent BJ cells compared to the proliferating cells (compare lanes 'L' for proliferating and replicatively senescent cells in Figure 1B). The increased cell surface protein level in replicatively senescent cells was verified by comparing the amount of eluted biotinylated L1CAM from streptavidin matrix (see lines 'E' in Figure 1B). This was further confirmed by flow cytometry analysis using live cell staining with L1CAM antibody, where the surface protein level of L1CAM was markedly elevated in replicatively senescent BJ cells (Figure 1C). Note, similar cell surface enhancement of L1CAM in replicative senescent BJ cells was observed also for fibronectin and PVR (Supplementary Figure 1E). The L1CAM protein elevation was concordant with the changes of the L1CAM mRNA levels (Figure 1D and Supplementary Figure 1C, D). Using indirect immunofluorescence, we observed marked heterogeneity of L1CAM cell surface levels among individual cells in both populations of proliferating and senescent BJ cells (Figure 1E; for validation of antibody specificity, see Supplementary Figure 1F, G; note that the L1CAM antibody staining of formaldehyde-fixed cells was nonspecific).

Altogether, our data show that L1CAM is overrepresented on the cell surface of replicatively senescent BJ fibroblasts; and L1CAM’s enhanced surface protein level corresponds to the increased expression of *L1CAM* mRNA.

### L1CAM expression is cell type- and senescence stimulus-dependent

**Figure 2:**
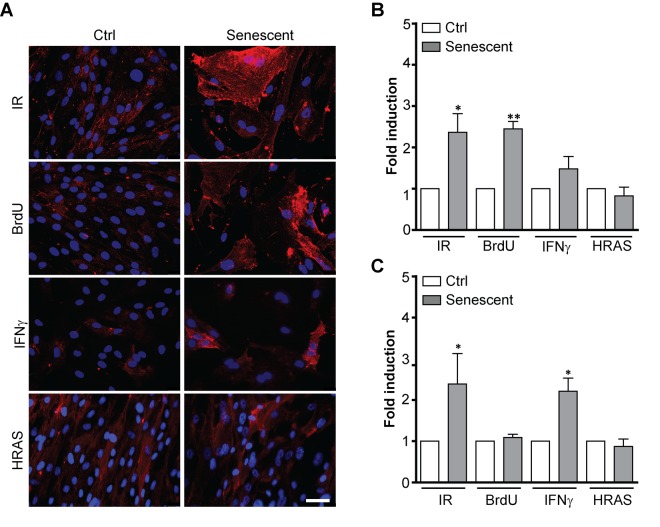
**L1CAM expression in premature senescence induced by various stimuli.** BJ fibroblasts were brought to premature senescence by γ-irradiation (PD 32, IR 20 Gy), 100 μM 5-bromo-2'-deoxyuridine (PD 32, BrdU), 500 U/ml IFNγ (PD35), or by induction of oncogenic HRAS using the Tet on system (see Materials and Methods). Cell surface expression of L1CAM estimated by live cell immunostaining with L1CAM antibody was detected microscopically (**A**) or (**B**) by FACS. The values representing three independent experiments are shown as a fold induction relative to control. (**C**) Real time RT-qPCR quantification of mRNA levels of L1CAM in BJ cells brought to premature senescence as in A. The values representing three independent experiments are shown as a fold induction relative to control. GAPDH was used as a reference gene. For statistics, two-tailed Student’s t-test was used; p ˂ 0.05 (*); p ˂ 0.01 (**); p ˂ 0.001 (***). Scale bar, 50 μm.

During serial cultivation of cells *in vitro* there is a likelihood of selection of clones with enhanced replicative potential [41]. To examine whether increased L1CAM expression in replicatively senescent BJ cells was due to clonal selection of cells bearing higher L1CAM expression, we followed the expression of L1CAM in a scenario of prematurely induced senescence in BJ cells triggered by ionizing radiation (IR) [42], 5-bromo-2'-deoxyuridine (BrdU) [43], and interferon-γ (IFNγ) [16,44], or overexpression of oncogenic H-Ras(V12) [46]. With the exception of H-Ras-induced senescence, the cell surface expression of L1CAM was increased in BJ fibroblasts upon exposure to all other stimuli (Figure 2A, B; see Supplementary Figure 1A for SA-β-gal staining), indicating that cell surface expression of L1CAM is not the result of a clonal selection during serial passaging. The lack of L1CAM induction in H-Ras oncogene-induced senescence suggested the dependence of L1CAM expression on the type of senescence-inducing stimulus. Moreover, we observed that the *L1CAM* transcript level remained unchanged after BrdU treatment despite enhanced L1CAM cell surface expression (Figure 2C), indicating that both *de novo* synthesis and/or enhanced (re)localization of L1CAM to the cell surface can take part in a mechanism of its enhanced cell surface expression. The heterogeneity of L1CAM expression in the population of SC was apparent among prematurely senescent cells as well.

To determine whether the heterogeneous expression of L1CAM in senescent cells stems from clonal heterogeneity present already in proliferating BJ cells, we sorted proliferating BJ cells according to their surface L1CAM level by FACS to populations with low (L1CAM^low^) and high (L1CAM^high^) expression (Supplementary Figure 2A) and followed the L1CAM levels for several population doublings. Notably, the differences in L1CAM levels between the sorted subpopulations balanced out after approximately ten population doublings (Supplementary Figure 2B) indicating that epigenetic rather than genetic factors likely determine the L1CAM heterogeneity. No differences in proliferation of L1CAM 'high' versus 'low' cells were observed (Supplementary Figure 2C), consistent with the notion that L1CAM expression is not linked with proliferation advantage of any subpopulation. In addition, there were no significant differences in the occurrence of DNA damage foci (detected as 53BP1 and serine 139 phosphorylated histone H2A.X) between L1CAM^high^ and L1CAM^low^ cells (see Supplementary Figure 2D and E). We also did not observe any marked morphological differences among senescent L1CAM^high^ and L1CAM^low^ populations of BJ cells, except that L1CAM^high^ cells were slightly larger in size (mean area ± SEM: 327.6 ± 5.317 μm^2^ for L1CAM^low^ and 344.9 ± 4.113 μm^2^ for L1CAM^high^; two-tailed paired test, p = 0.0123; Supplementary Figure 2F). Furthermore, down-regulation of L1CAM in replicatively senescent cells did not result in escape of cells from senescence (Supplementary Figure 2G), indicating that L1CAM is not involved in senescent cell cycle arrest.

**Figure 3:**
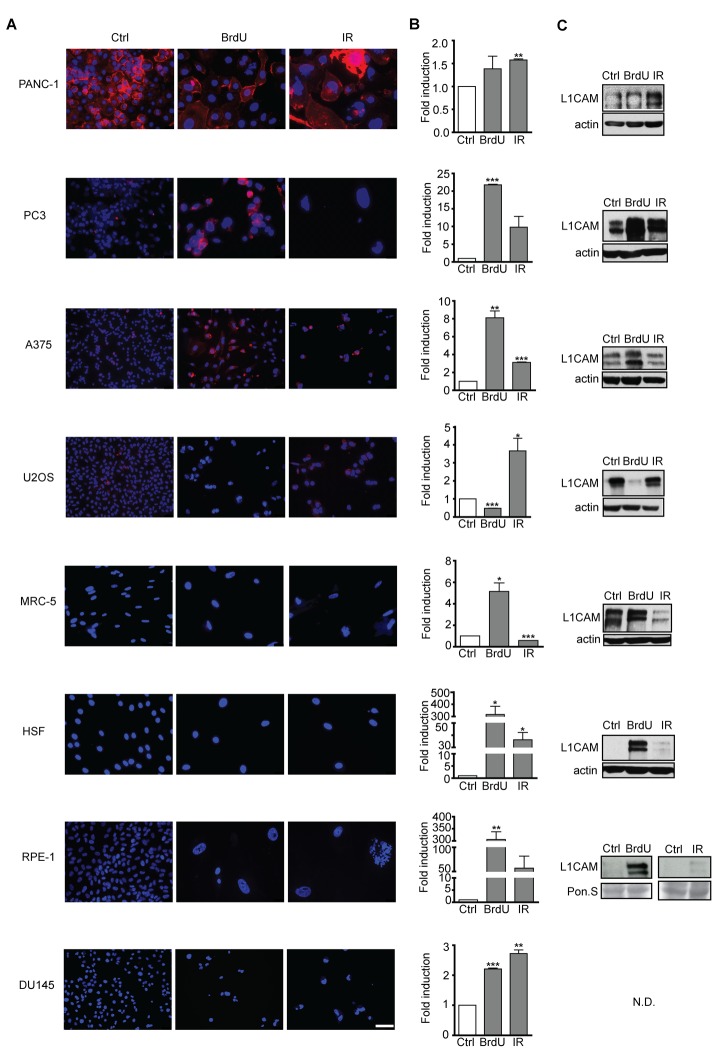
**L1CAM expression in normal and tumor senescent cells depends on the cell type and senescence-inducing stimulus**. Normal (MRC5, HSF), immortalized (RPE-1) and tumor (PANC-1, PC3, A375, U2OS, and DU145) cells were brought to senescence either by BrdU (10 μM for A375, 100 μM for rest of cell types) or IR (10 Gy) and assayed for cell surface L1CAM protein expression by live cell immunostaining with L1CAM antibodies. (**A**) L1CAM mRNA expression by real time RT-qPCR quantification (**B**) and total L1CAM protein level by immunoblotting (**C**). GAPDH was used as a reference gene; β-actin was used as a loading control. The values representing two independent experiments are shown as a fold induction relative to control. N.D., not detected. Scale bar, 50 μm.

Next we tested a panel of other human cell types, including normal and cancerous cells, for L1CAM expression during the development of premature senescence. Figure 3 summarizes L1CAM mRNA, total and cell surface protein levels in a variety of cell types brought to senescence by IR or BrdU (Supplementary Figure 3A). In accord with a previous study [47], pancreatic carcinoma PANC-1 cells featured high L1CAM cell surface expression even under unperturbed conditions (see Supplementary Figure 3B for the relative transcript level of L1CAM in all cell types analyzed). IR but not BrdU induced both L1CAM transcript level and cell surface expression in PANC-1 and similarly in osteosarcoma U2OS cells (Figure 3A, B). In contrast, BrdU induced L1CAM expression in prostate cancer PC3 and melanoma A375 cells more potently than IR. Normal diploid fibroblasts MRC-5 and HSF-1, and immortalized retinal pigment epithelial RPE-1 cells responded by elevation of L1CAM mRNA and total protein levels in dependence on the stimulus but without concomitant protein expression on the cell surface, indicating again that cell surface exposure of L1CAM is regulated. Note that the expression of L1CAM is the lowest in RPE-1 among the tested cell lines (Supplementary Figure 3B); therefore, it is possible that the observed elevation of the L1CAM level after senescence induction was not sufficient for detection of the surface protein level. The transcript levels of L1CAM were only slightly induced in prostate cancer DU145 cells exposed to either IR or BrdU; and the L1CAM protein remained undetectable (Figure 3C). In general, our data showed that higher levels of L1CAM transcriptional induction appear to be necessary for the increase of the total protein level; however, even an increased total protein level is not always expressed as the enhanced presence of protein on the cell surface.

To investigate whether the L1CAM is also induced during senescence in different species, we triggered premature senescence in three mouse cell lines TRAMPC2, TC1 and B16F10 by exposure to docetaxel (7.5 μM, 4 days) as reported previously [48,49]. In all three cell lines, L1CAM transcript levels were elevated after docetaxel treatment (see Supplementary Figure 4A for L1CAM level and Supplementary Figure 4B for senescence-associated beta-galactosidase staining) indicating that L1CAM induction during senescence is not restricted to human cells.

To conclude, the senescence-associated expression of L1CAM on the cell surface is feature shared in normal and cancerous cells of human and mouse origin, but dependent on cell type and senescence-inducing stimulus. Our findings also indicate that L1CAM cell surface expression is a complex process regulated both at the level of transcription and posttranscriptionally.

### L1CAM expression is a downstream event linked to inhibition of cyclin-dependent kinases by p16ink4a

**Figure 4:**
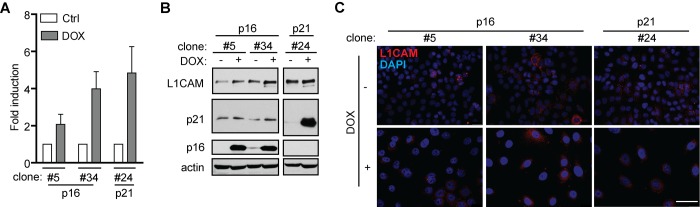
**L1CAM expression is a downstream event linked to inhibition of cyclin-dependent kinases.** L1CAM mRNA (**A**), total protein (B) and surface expression in control cells (ctrl; DOX-) and doxycycline-induced (DOX+) H28 cell clones #5, #34 (expressing p16) and #24 (expressing p21). L1CAM mRNA level was normalized to GAPDH. All experiments were performed in three independent replicates. Scale bar, 100 μm.

Next, to decipher the mechanisms mediating L1CAM expression during induction of senescence, we first investigated to which phase of the cell cycle checkpoint cascade the expression of L1CAM is linked. To uncouple the inhibition of cell cycle from upstream events of DNA damage response we utilized ectopic expression of tetracycline-regulatable inhibitor of cyclin-dependent kinases p16^ink4a^ (p16) in human mesothelioma H28 cells in comparison to ectopic expression of p21^waf1^ (p21) known to induce DNA damage response [50]. Doxycycline induction of both p16 and p21 in two single cell-derived clones (p16) and one single-derived p21 clone led to cell cycle arrest (Supplementary Figure 5A; for senescence-associated β-galactosidase staining, see Supplementary Figure 5B), as observed previously [51]. In contrast to p21, no signs of enhanced DNA damage response detected as 53BP1/γH2AX foci were observed after p16 induction when compared to non-induced cells (Supplementary Figure 5C). A reproducible increase of L1CAM mRNA (Figure 4A) and protein (Figure 4B) was observed after induction of both p16 (Figure 4C) and p21 (not shown), however, without detectable increase of the L1CAM cell surface protein level. These findings indicated that the induction of L1CAM expression is a downstream event following induction of cdk protein inhibitor(s).

### Cell type-dependent and mutual interaction of L1CAM and Erk signaling pathways

**Figure 5:**
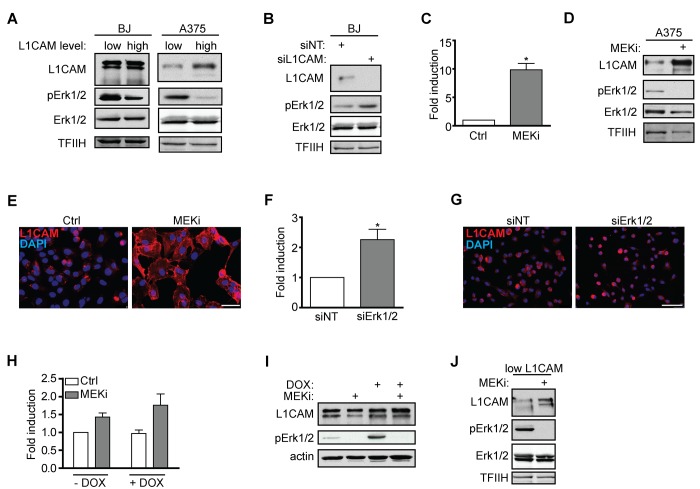
**Interaction of L1CAM with the Erk signaling pathway.** (**A**) Erk 1/2 activity detected as phosphorylation of Erk1/2 (pErk1/2) compared in BJ and A375 cells sorted for L1CAM high and low cell surface level. (**B**) The effect of L1CAM downregulation using RNA interference on Erk1/2 activity detected by immunoblotting in BJ fibroblasts. (**C**) L1CAM mRNA level estimated by real time RT-PCR after inhibition of MEK by selumetinib (10 μM; MEKi) in A375 cells. Total (**D**) and surface L1CAM levels (**E**) detected by immunoblotting and live cell staining, respectively, in A375 cells after MEK inhibition using selumetinib (10 μM; MEKi). L1CAM mRNA (**F**) and surface protein level (**G**) in A375 after downregulation of Erk1/2 using RNA interference (siErk1/2). L1CAM mRNA (**H**) and total protein (**I**) levels in control (-DOX) and H-RAS-induced (+DOX) BJ cells before (ctrl) and after inhibition of MEK using selumetinib (10 μM; MEKi). (**J**) The effect of MEK inhibition by selumetinib (10 μM; MEKi) on the L1CAM total protein level in H-RAS-induced senescent BJ cells sorted for low L1CAM level. Non-template siRNA was used as a control (siNT). For immunoblotting, TFIIH or β-actin were used as a control of equal protein loading. For real time RT-PCR, GAPDH was used as the reference gene. Scale bar, 100 μm. All experiments were performed in three independent replicates. p ˂ 0.05 (*), two-tailed Student’s t-test.

It has been reported that activation of the L1CAM signaling pathway by antibody-mediated L1CAM crosslinking or manipulation with its level affect the activity of Erk1/2 [52–54]. Indeed, we observed that the basal activity of Erk1/2 was higher in L1CAM^low^ human melanoma A375 and BJ cells when compared to L1CAM^high^ cells (Figure 5A). Additionally, knockdown of L1CAM in BJ cells increased Erk1/2 phosphorylation (Figure 5B). This data obtained in unperturbed cell culture conditions underscored the role of L1CAM in the regulation of activity of Erk1/2.

The other way around, inhibition of Erk1/2 activation by selumetinib, a chemical inhibitor of MEK, in A375 (Figure 5C) and HeLa (data not shown) cells resulted in marked elevation of L1CAM mRNA, total and cell surface protein expression (Figure 5D, E), which indicated that the MAPK pathway may operate in a negative feedback loop to suppress L1CAM, thus balancing its own activity. Further, combined knockdown of Erk1 and/or Erk2 by RNA interference resulted in elevation of L1CAM mRNA (Figure 5F) and surface protein expression (Figure 5G), further supporting the interplay between L1CAM and Erk pathways. Nevertheless, the exposure of BJ cells to selumetinib did not show any effect on L1CAM mRNA and protein levels (not shown), indicating cell type-specific regulation of the L1CAM expression.

These findings fit well with the absence of L1CAM expression during H-RAS oncogene-induced senescence (OIS). To exclude the possibility that our strain of BJ fibroblasts with tetracycline-inducible RAS oncogene lost the competence to induce L1CAM (due to cloning of cells with aberrant L1CAM regulation), we exposed Ras-induced senescent BJ cells presorted for low level of L1CAM to MEK inhibitor selumetinib. As shown in Figure 5J, the inhibition of MEK resulted in an increased L1CAM protein level, indicating unperturbed sensitivity of L1CAM to Erk1/2 inhibition in OIS cells, supporting the suppressive role of the Ras/MAPK pathway on L1CAM expression.

Overall, our results indicate a crosstalk between L1CAM and Ras/MAPK pathways, where the elevated level of L1CAM is associated with lower Erk1/2 activity and the Ras/MAPK signaling exerts a suppressive effect on L1CAM gene expression.

### L1CAM expression is linked to metabolic changes

**Figure 6:**
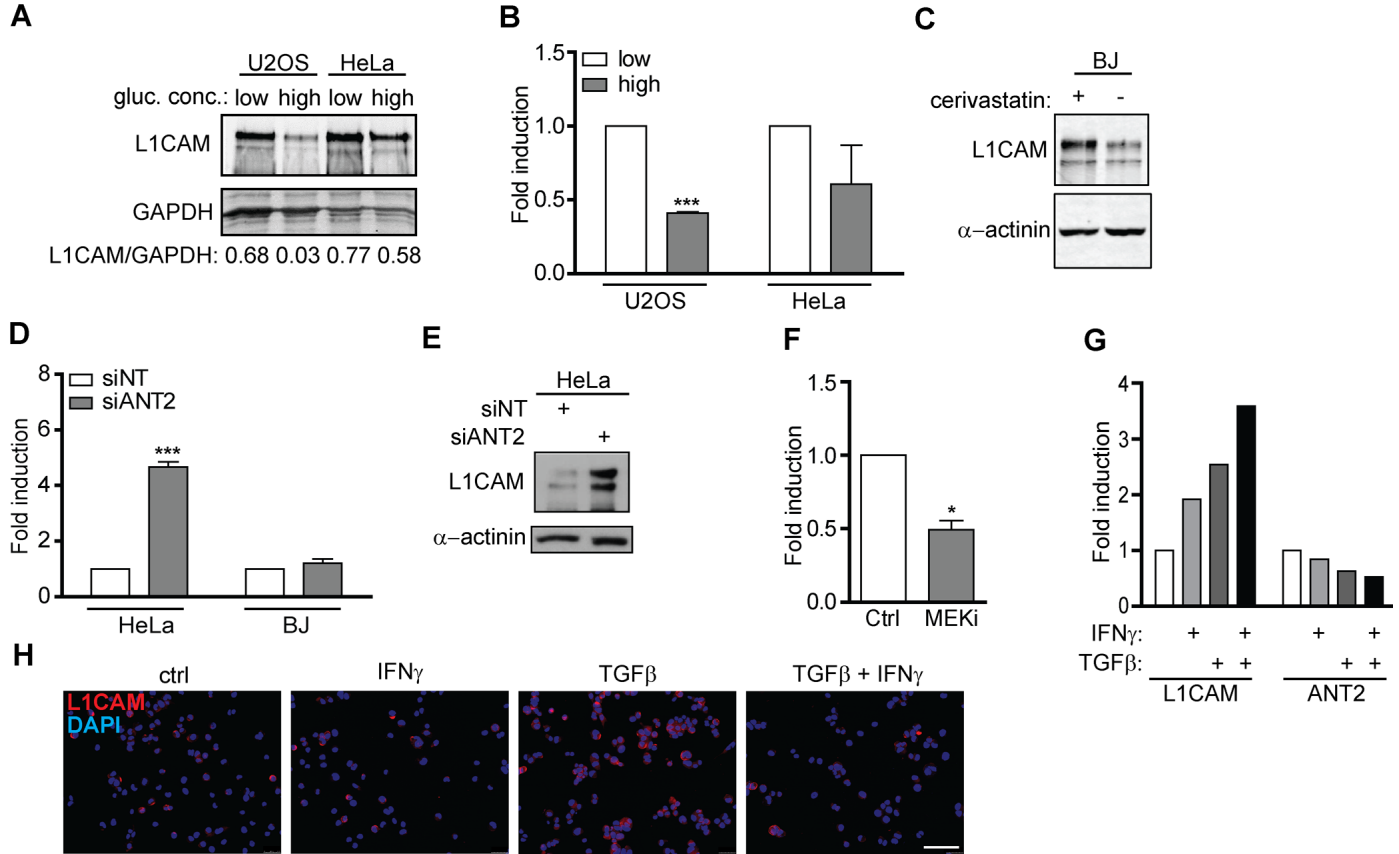
**L1CAM expression is linked to metabolic changes.** The effect of high (4.5 g/L) and low (1.0 g/L) glucose concentration in cultivation medium on total L1CAM protein (**A**) and L1CAM mRNA levels (**B**) in U2OS and HeLa cells detected by Western blotting and real time RT-PCR, respectively. (**C**) The effect of inhibition of the mevalonate pathway by cerivastatin on the L1CAM total protein level in BJ fibroblasts. L1CAM mRNA in HeLa and BJ cells (**D**) and total L1CAM protein in Hela cells (**E**) after downregulation of ANT2 using RNA interference. (**F**) ANT2 mRNA level after inhibition of MEK by selumetinib (10 μM; MEKi) in A375 cells. L1CAM and ANT2 transcripts (**G**) and L1CAM surface expression (**H**) in A375 cells exposed to 500 U/ml IFNγ, 10 ng/ml TGFβ, or their combination for 4 days. For real time RT-qPCR experiments, GAPDH was used as the reference gene. For immunoblotting, GAPDH or α-actinin were used as loading controls. p ˂ 0.05 (*); p ˂ 0.01 (**); p ˂ 0.001 (***), two-tailed Student’s t-test. Scale bar, 100 μm. All experiments were performed in three independent replicates.

Given that *L1CAM* transcription is regulated by hypoxia in tumor cells [55], we asked whether metabolic changes associated with senescence (see e.g. references [56–58]) contribute to regulation of *L1CAM* gene expression. We noted that the concentration of glucose in culture medium affected the expression of *L1CAM* in U2OS and HeLa cells. Both cell types cultured in higher concentration of glucose (4.5 g/L) expressed lower levels of L1CAM compared to cells cultured in 1 g/L glucose, indicating that the expression of L1CAM might be linked to glycolytic energy metabolism of tumor cells (Figure 6A, B). Furthermore, the level of *L1CAM* transcript and protein increased after inhibition of the mevalonate pathway (chosen here for being one of the pathways that utilize acetyl-CoA, derived from glucose metabolism) in BJ fibroblasts by cerivastatin (Figure 6C). As reported previously, various forms of cellular senescence are accompanied by repression of solute carrier family 25 member 5 (SCL25A5) coding for ADP/ATP translocase 2 (ANT2 [59,60];), which has been implicated in glycolytic metabolism of tumor cells [61–63]. RNA interference-mediated downregulation of ANT2 but not ANT3 (Supplementary Figure 6A) in HeLa and BJ cells led to marked elevation of L1CAM both at mRNA and protein levels (Figure 6E). The intrapopulation heterogeneity of L1CAM expression was diminished after ANT2 knockdown (data not shown). Interestingly, the inhibition of MEK by selumetinib in A375 cells resulted in suppression of the ANT2 mRNA level associated with the increase of L1CAM expression (see Figure 5C and Figure 6F), suggesting a reciprocal link between ANT2 and L1CAM expression. In support of this notion, there was also an inverse relationship between the mRNA levels of both genes in A375 cells exposed to IFNγ and TGFβ (Figure 6G), the cytokines reported to induce senescence [16,60]. Note that TGFβ alone strongly induced cell surface expression of L1CAM in A375 cells (Figure 6H), which was associated with stronger cytostatic effect compared to IFNγ (Supplementary Figure 6B).

Altogether our data indicate that the induction of L1CAM during senescence might be linked to accompanying metabolic changes, specifically to suppression of the *SCL25A5/ANT2* gene expression.

### L1CAM increases cell migration and adhesion of both proliferating and senescent BJ cells

**Figure 7:**
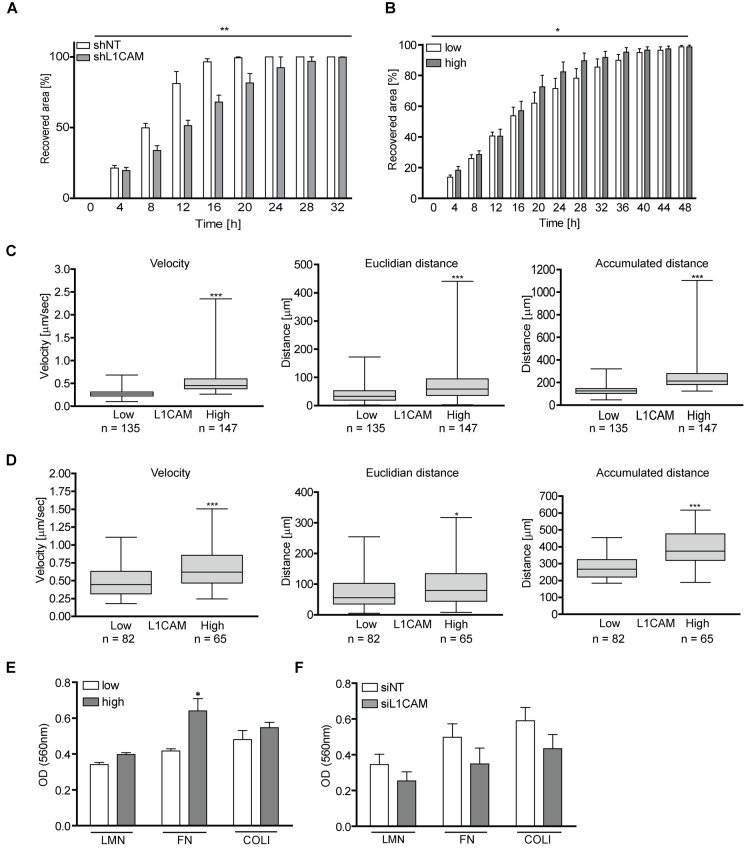
**L1CAM levels correlate with enhanced cell migration and adhesion both in proliferating and senescent cells.** (**A**) Wound healing assay of proliferating BJ after RNA interference-mediated knockdown of L1CAM and (**B**) replicatively senescent BJ fibroblasts sorted according to cell surface expression of L1CAM. (**C**) 3D migration assay of replicatively senescent BJ sorted according to L1CAM cell surface expression presented as velocity (left chart), Euclidian distance (middle chart) and accumulated distance (right chart). (**D**) 3D migration assay of unsorted replicatively senescent BJ. Left chart – velocity; middle chart – Euclidian distance; and right chart – accumulated distance. (**E**) Adhesion assay of replicatively senescent BJ fibroblasts sorted according to cell surface L1CAM expression and (F) after L1CAM knockdown. All experiments were performed in three independent replicates. p ˂ 0.05 (*); p ˂ 0.01 (**); p ˂ 0.001 (***), two-tailed Student’s t-test.

Several studies reported the role of L1CAM in enhanced migration of tumor cells (see, e.g., references [31,64]). Indeed, in a wound healing assay, young BJ cells with downregulated L1CAM using lentivirally transduced short hairpin RNA recovered the disrupted area significantly slower than control cells (Figure 7A), indicating that migration of normal cells can also be affected by L1CAM surface expression (see reference [54].). Next we asked whether enhanced expression of L1CAM is linked with the migratory properties of senescent cells. Utilizing heterogeneity of L1CAM expression in the cell population of BJ senescent cells, we tested migration of BJ cells sorted by FACS according to L1CAM cell surface expression. Replicatively senescent BJ cells sorted for higher surface L1CAM levels healed the scratch more efficiently than their counterparts with lower cell surface expression of L1CAM (Figure 7B). To further corroborate these findings, we employed a 3D migration assay to follow cell motility induced by fetal bovine serum as attractant. Using time-lapse cell tracking, several parameters such as velocity, Euclidean distance (length of migration that the cell overcomes directly from the start point to the endpoint), and accumulated distance (cell’s trajectory) were evaluated. Notably, senescent BJ cells with high L1CAM cell surface expression migrated faster and covered longer Euclidean and accumulated distances (Figure 7C). To exclude the possibility of an impact of growth conditions on the tested subpopulations, a 3D random migration assay of non-sorted but L1CAM antibody-labeled senescent BJ fibroblasts was also performed. The results revealed a similar trend as in the case of L1CAM-sorted BJ cells. L1CAM^high^ BJ cells migrated faster and travelled further distances (Figure 7D) compared to L1CAM^low^ BJ cells. The migration of proliferating BJ fibroblasts in the wound healing assay was faster compared to replicatively senescent cells, where young BJ covered the disrupted area approximately three times faster (by 16 hours) compared to senescent cells (48 hours; see references [65,66]). The proliferation of young cells contributed to faster wound recovering as well; however, the difference in the velocity was significant (Supplementary Video 1 and Supplementary Video 2).

Next we asked whether the cell surface level of L1CAM correlates with the cell adhesive properties. As shown in Figure 7E, replicatively senescent L1CAM^high^ BJ cells showed 10, 17 and 20% higher adhesion to the components of extracellular matrix laminin, fibronectin, and collagen 1, respectively, compared to senescent L1CAM^low^ BJ cells. To confirm the contribution of L1CAM to cell adhesive properties, L1CAM was downregulated in senescent BJ fibroblasts by RNA interference (Figure 7F; for the level of L1CAM downregulation, see Supplementary Figure 6C). Indeed, control cells adhered more efficiently compared to the cells with a decreased L1CAM level.

Taken together, the enhanced level of L1CAM correlates with increased migratory properties not only of proliferating but also senescent BJ cells. The expression of L1CAM affects the adhesion of BJ cells to the components of extracellular matrix.

## DISCUSSION

Despite that cellular senescence is a phenomenon well characterized and recognizable *in vitro* and the detection of SC in tissues is now less problematic due to the recently published robust method for their detection [67], there is a lack of strictly specific markers that would unambiguously allow targeting of SC for therapeutic purposes [68]. In search of cell-surface determinants overrepresented on senescent cells, we identified adhesion molecule L1CAM as being enhanced on replicatively senescent BJ fibroblasts. Further analysis of L1CAM expression in different senescence-inducing scenarios revealed that L1CAM expression depends on cell type and senescence-inducing stimulus, it shows marked intra-population heterogeneity and multiple levels of control, including regulation of the cell surface exposure. Our data also show that the L1CAM signaling pathway is interlinked with Erk signaling in a reciprocal manner. Stimuli changing cellular metabolism such as glucose levels, inhibition of the mevalonate pathway, and suppression of ANT2 (SCL325A5) all affected the level of L1CAM expression, suggesting that *L1CAM* gene expression might be controlled by the metabolic changes accompanying the development of cellular senescence. The L1CAM level correlated with increased migratory properties of both proliferating and senescent BJ cells and with enhanced adhesion of cells to the components of extracellular matrix.

Our proteomic comparative analysis of cell surface proteins of proliferating and senescent BJ cells based on surface protein biotinylation revealed more than 70 proteins significantly enhanced on the plasma membrane of senescent cells. In previous studies using different methodological approaches, several of these proteins, such as tissue inhibitor of metalloproteinase 3 (TIMP3) [38], γ-glutamyltranspeptidase 2 (GGT2) [39] and ADAMTS1 [40] have already been reported as being overrepresented in senescent cells, thereby validating the technique employed in this study.

The L1CAM/L1 cell adhesive molecule is a transmembrane glycoprotein originally discovered as a cell adhesion molecule in the nervous system [69], where it plays a crucial role during the brain development, being specifically involved in the neurite outgrowth [70] and fasciculation [71], adhesion of neurons and astrocytes [72], and cell migration [73]. L1CAM attracted our attention due to its aberrant expression in several types of human tumors, where it acts as a promalignant factor and progression marker. For instance, L1CAM expression increases with progression of breast cancer [31]. Further, L1CAM promotes tumor progression and metastasis in melanoma [32] and ovarian [33], gastric [34], lung [35], and pancreatic cancers [36,37], where its presence is associated with a poor prognosis. Besides promoting cancer cell migration (see, e.g., [36]), including perineural invasion of cancer cells (for a review, see ref [74].), L1CAM confers anti-apoptotic protection, chemoresistance [75] and radioresistance [76], stimulates cell survival [77] and acts as a pro-angiogenic factor [78].

Analysis of L1CAM induction in various cell types and senescence conditions revealed that the increase of the L1CAM total protein level during senescence was mostly associated with the increase of *L1CAM* transcript, indicating senescence-induced *de novo* transcription. Nevertheless, as in the case of HSF and RPE-1, the elevation of the total L1CAM protein must not necessarily be accompanied by the increase of its mRNA level indicative of senescence-mediated posttranscriptional regulation of L1CAM. Moreover, the increase of the L1CAM total protein level was not always associated with its enhanced cell surface presence, indicating that the exposure of L1CAM on the cell surface of senescent cells is a complex process involving regulation at the transcriptional, posttranscriptional, and posttranslational levels.

Regulation of the *L1CAM* gene expression is controlled by diverse stimuli such as hypoxia or TGFβ signaling. It has been shown that inhibition of HIF-1 leads to inhibition of L1CAM and subsequent blockage of tumor growth and metastasis in lung cancer [55].

The cell cycle block responsible for premature senescence provoked by BrdU or IR is mediated by DNA damage response-induced expression of p21 and/or p16. In our attempt to understand the mechanism of L1CAM induction in premature senescence we found out that artificial expression of p16 itself is capable of elevating *L1CAM* transcript and protein levels, indicating that upstream DNA response signaling is not a prerequisite for senescence-mediated transcriptional induction of *L1CAM*. The notion that induction of *L1CAM* transcription was entirely absent in oncogenic Ras-induced senescence (Figure 2) led us to investigate the role of Ras/MAPK signaling in the control of *L1CAM* gene expression. Several reports point to the role of L1CAM signaling in augmentation of Erk activity ([52–54]). Based on observed intrapopulation variability where the L1CAM levels inversely correlated with Erk1/2 'basal' activity which increased after L1CAM downregulation, our results showed rather opposite relationship indicating cell type differences. In contrast, chemical inhibition of MAP kinase MEK or downregulation of Erk1 or Erk2 resulted in elevation of the L1CAM transcript and protein levels. This suggests the suppressive role of the Ras/MAPK pathway on the L1CAM expression and the existence of a regulatory circuit controlling the mutual balance of L1CAM and the Ras/MAPK signaling, where the expression of L1CAM is controlled by the Erk pathway in a negative feedback loop. It is therefore likely that the level of L1CAM expression in a particular cell type depends on the equilibrium of interlinked activities of Erk and L1CAM pathways. Furthermore, we found that L1CAM expression was also upregulated in A375 cells exposed to TGFβ in concordance with previous reports [79,80]. Multiple interactions between TGFβ/SMAD and the Ras/MAPK pathways have been described (reviewed, e.g., in [81]). For instance, the Ras/MAPK pathway can suppress the activity of SMAD3 by Erk-dependent phosphorylation of SMAD3 [82]. It is therefore likely that chemical inhibition of MEK relieves the Ras/MAPK-mediated inhibition of the TGFβ pathway, resulting in increased expression of L1CAM; however, this warrants further investigation. The reciprocal expression of *SCL25A5/ANT2* and *L1CAM* genes could reflect their reverse transcriptional regulation by the TGFβ pathway. The *ANT2* gene belongs to early immediate genes induced by mitogens such as platelet-derived growth factor and epidermal growth factor [83], indicating the role of the MAPK pathway in *ANT2* gene expression. Expression of ANT2 is strictly growth-dependent, and ANT2 is suppressed during growth cessation mediated by TGFβ-activated NFI/SMAD transcription factor complexes [59,84]. In contrast, L1CAM expression is induced by TGFβ ([79,80] and this study) and suppressed by Ras/MAPK signaling (Figure 5). However, it seems that L1CAM expression might be ANT2-dependent, as RNA interference-mediated knockdown of ANT2 resulted in L1CAM induction. As L1CAM is induced by metabolic signals, we propose that changes in the energy metabolism in response to the cell cycle arrest induce L1CAM. Further study is needed to reveal the mechanism of L1CAM induction in SC.

The cause of heterogeneous expression of L1CAM in proliferating and senescent cell populations *in vitro* is unclear. We observed that cells sorted according to the L1CAM surface level sustained its level of expression for several consecutive population doublings prior the establishment/return to the original heterogeneity, indicative of the role of epigenetic rather than genetic factors (clonal evolution) in the L1CAM intra-population heterogeneity. Based on our results we propose that the individual cell-specific sensitivity to Ras/MAPK and TGFβ signaling sets the level of the L1CAM surface expression.

In accord with previous study showing that manipulation of the level of L1CAM affects the migration properties of breast cancer cells [31], we observed that downregulation of L1CAM attenuates migration of proliferating BJ cells, supporting the role of L1CAM in cell locomotion. Intriguingly, also SC with a higher level of L1CAM surface expression migrated faster, which was accompanied by increased adhesion to extracellular matrix proteins laminin-1, collagen-1, and fibronectin. Senescent cells migrated significantly slower compared to proliferating cells, which may reflect overall changes in the adhesion apparatus and cytoskeleton of senescent cells, and likely an aberrant cell polarity and response to chemotactic stimuli. We attempted to decipher the mechanism of enhanced locomotory properties of cells with elevated L1CAM. To this end, we analyzed the number and shape of focal adhesions, the RhoA activity, integrin α2β1 and integrin αV levels, and the cell polarization to a wound; however, we did not find any significant differences among L1CAM high and low cell subpopulations (see Supplementary Figure 7A, B, C, D, E, and F). Our results are also broadly consistent with the L1CAM participation in axon guidance and neuronal migration, in a pathway culminating in MEK and Erk activation [85]. Furthermore, a very recent study showed that ionizing radiation reduces ADAM10 expression in brain microvascular endothelial cells and this indirectly leads to enhanced L1CAM protein level [86], suggesting yet another level of stress-responding L1CAM regulation.

Senescent cells represent premalignant cellular forms [6], and the expression of the L1CAM oncoprotein in senescent cells may provide them with specific features that can be inherited by tumor cells bypassing the senescence state [87]. There is accumulating evidence supported by experimental animal models that cancer cells can disseminate early during tumor development to form metastases later, after a shorter or longer period of dormancy (see, e.g [88,89], for a review, see [90]), sometimes even without observable formation of a primary tumor. Although it has not yet been proved experimentally that senescent cells can spread to distant tissues from the site of their origin, such possibility cannot be ruled out. Provided cellular senescence may be bypassed, as supported by several experimental findings (see, e.g., references [91,92]), the migratory and invasive properties of senescent cells might become important. Based on the correlation between the L1CAM level and tumor invasiveness it cannot be excluded that migration of senescent cells in the tissues might also be affected by L1CAM expression. Given the emerging roles of SC in promoting both tumorigenesis and organismal aging, our present results may inspire future senolytic approaches through exploiting the cell-surface expression of L1CAM in cellular senescence.

## MATERIALS AND METHODS

### Chemicals and antibodies

Laminin-1 (human fibroblast-derived) and fibronectin (human plasma) were obtained from Sigma-Aldrich (St. Louis, MO, USA). Rat tail collagen type I was purchased from Millipore. Recombinant IFNγ was purchased from Peprotech (Rocky Hill, NJ, USA). Mouse monoclonal antibody against L1CAM from Sigma-Aldrich (Sigma-Aldrich), mouse monoclonal antibody against phosphoserine 139 of histone H2AX (Millipore, Billerica, MA, USA), rabbit polyclonal antibody against 53BP1 (Santa Cruz, CA, USA), and rabbit polyclonal antibody against γ-tubulin (Sigma Aldrich) were used for indirect immunofluorescence. Mouse monoclonal antibody against GAPDH (GeneTEX), rabbit polyclonal antibody against pan-actin (Sigma-Aldrich, St. Louis, MO, USA), and rabbit polyclonal against αTFIIH (Santa Cruz), rabbit polyclonal antibody against integrin αV (Cell Signaling), and mouse monoclonal antibody against integrin α2β1(Abcam) were used for immunoblotting. Anti-mouse IgG antibody Alexa 555 (Invitrogen, Carlsbad, CA, USA) and anti-rabbit IgG antibody Alexa 488 (Invitrogen) were used as secondary antibodies.

### Cell cultures and senescence induction

Human cancer cell lines (PC3 (ATCC^®^ CRL-1435^™^), DU145 (ATCC^®^ HTB-81^™^), A375 (ATCC^®^ CRL-1619^™^), U2OS (ATCC^®^ HTB-96^™^), PANC-1 (ATCC^®^ CRL-1469^™^)) and immortalized human epithelial cells (hTERT RPE-1, ATCC^®^ CRL-4000^™^) were cultured in Dulbecco’s modified Eagle’s medium (D-MEM, Thermo Fisher Scientific, Waltham, MA, USA) supplemented with 10% fetal bovine serum (FBS, Gibco/Thermo Fisher Scientific) and 4.5 g/l of glucose. Primary human diploid fibroblast BJ (ATCC^®^ CRL-2522^™^, population doubling 25-85), MRC5 (ATCC^®^ CCL-171^™^, population doubling 24-61), and human skin fibroblasts (HSF-1; population doubling 20 - 26) were cultured in Dulbecco’s modified Eagle’s medium (D-MEM, Thermo Fisher Scientific) supplemented with 10% fetal bovine serum (FBS, Gibco/Thermo Fisher Scientific) and 1 g/l of glucose. Both cell culture media were supplemented with penicillin/streptomycin (Sigma-Aldrich). Cells were plated to match the equal cell density of control and senescent cell culture at the time of harvest.

Cells were kept at 37°C under 5% CO_2_ atmosphere and 95% humidity. To bring cells to replicative senescence, BJ cells were split in ratio 1: 2 until proliferation exhaustion (population doubling 85). To induce premature senescence, cells were treated either with 100 µM BrdU (BJ, MRC5, HSF-1, RPE, PC3, DU145, U2OS, PANC-1) for 10 days or with 10 µM BrdU (A375) for 7 days, 500 U/ml IFNγ (BJ) for 21 days, or irradiated with a dose of 10 or 20 Gy, as indicated. Oncogenic mutant H-Ras^V12^ expressed in BJ was prepared as described earlier [93,94]. H-Ras was induced by addition of 2 μg/ml of doxycycline every 48 hours for 16 days.

### Preparation of tet-one p16 and p21 constructs

cDNA sequences for p16 and p21 were synthesized in Genescript. The cDNAs were subcloned to the Lenti-X™ Tet-One™ Inducible Expression System, which was obtained from Clontech (631847).

### Preparation of recombinant lentiviruses, transduction of cells and clonal expansion

Recombinant lentiviruses were obtained from calcium-phosphate transfected HEK 293T cells using packaging plasmids psPAX2 (Addgene, 12260) and pMD2.G (Addgene, 12259) together with either tet-one empty or tet-one p16 and p21 constructs. The medium containing lentiviral particles was harvested 36 to 48 h post-transfection, and the viral particles were precipitated using PEG-it (System Biosciences). Target cells H28 were transduced with viruses at multiplicity of infection MOI 5-10 and selected for puromycin resistance (2 μg/ml; Invivogen). For clonal expansion of each transduced cell type, single cells were sorted by BD FACSaria and the clones were then selected according to the expression profile of p16 or p21 protein.

### Senescence-associated-β-galactosidase assay

Cells grown on glass coverslips were fixed with 0.5% glutaraldehyde at room temperature for 15 minutes, washed with PBS supplemented with 1 mM MgCl_2_, and then incubated with pre-warmed X-gal solution (1 mg/ml X-gal (Sigma-Aldrich), 0.12 mM K_3_Fe[CN]_6_, 0.12 mM K_4_Fe[CN]_6_ × 3 H_2_O, 1 mM MgCl_2_ in PBS, pH 6.0) at 37°C and 5% CO_2_ for 4 to 24 hours (depending on the cell type) according to Dimri et al. [95]. After development of visible blue coloring inside the cells, coverslips were mounted in Mowiol containing 4',6-diamidino-2-phenylindole (DAPI; Sigma-Aldrich) and viewed by fluorescence microscope Leica DMRXA (Leica Microsystems, Germany) equipped with a color camera.

### Live cell immunofluorescence

Cells grown on glass coverslips were incubated with primary antibodies at room temperature for 15 minutes in PBS supplemented with Ca^2+^ and Mg^2+^ (PBS^+^). After washing with PBS^+^, incubation with secondary antibody was performed at room temperature for 15 minutes. After washing with PBS^+^, cells were fixed with 4% formaldehyde at room temperature for 15 minutes. Coverslips were mounted in Mowiol containing DAPI to counterstain nuclei and viewed by fluorescence microscope Leica DMRXA.

### Indirect immunofluorescence

Cells grown on glass coverslips were fixed with 4% formaldehyde and permeabilized with 0.1% Triton X-100 in two consecutive steps, each at room temperature for 15 minutes, and blocked with 10% FBS at room temperature for 30 minutes. After washing with PBS, cells were incubated with diluted primary antibodies at room temperature for 1 hour and then extensively washed with PBS/0.1% Tween 20. The incubation with secondary antibodies was performed at room temperature for 1 hour. Coverslips were mounted in Mowiol containing DAPI to counterstain nuclei and viewed by fluorescence microscope Leica DMRXA.

### Cell sorting

Cells were washed two times with PBS and detached from the cultivation plates by incubation with accutase (Accumax, Merck Millipore, USA) at 37°C and 5% CO_2_ for 2 to 3 minutes. To stop the protease, cultivation medium was added to the detached cells. Cell suspension was centrifuged at 700 x g for 5 minutes. The pellet was once washed with cultivation medium to get rid of the accutase, and the suspension was again centrifuged. Cell pellets were then stained with L1CAM antibody diluted 1: 100 in cultivation medium on ice for 20 minutes, centrifuged, washed twice with culture medium and incubated with secondary antibody (diluted 1: 500 in cultivation medium) on ice for 15 minutes. Cells were then washed twice with culture medium and after the last wash, they were resuspended in cultivation medium without serum and subjected to cell sorting.

### Cell cycle measurement

Cells were washed with PBS, trypsinized, and subsequently collected into fresh medium. After centrifugation (500 x g at 4°C for 3 min), the cell pellet was resuspended in PBS. For fixation, a suspension drop was let drop into a centrifuge tube with -20°C 100% ethanol while vortexing and kept in -20°C for at least 2 hours. Fixed cells were collected by centrifugation, the cell pellet washed with PBS, resuspended in PBS containing RNase A (final concentration 0.2 mg/ml; Thermo Fisher Scientific, Waltham, MA, USA) and incubated at RT for 30 min. Prior to FACS measurement, propidium iodide (final concentration 12.5 μg/ml)/NP40 (final concentration 0.1%) solution was added to the samples.

### Surface protein purification and mass spectrometry

BJ cells were labeled with ^13^C_6_ arginine. The surface proteins were isolated using the biotinylation method (according to manufacturer’s protocol, Thermo Fisher Scientific). Briefly, cells growing on a 10 cm^2^ dish were washed with ice-cold DPBS and incubated with 0.25 mg/ml sulfoNHS-S-S-biotin (Thermo Fisher Scientific) solution in DPBS at 4°C for 15 min. Cells were washed three times and the remaining unreacted NHS ester was quenched with 100 mM glycine in DPBS. After 10 min at 4°C, the unreacted NHS ester was washed away with DPBS. Cells were then lysed on the dish by adding 500 μl of lysis buffer (0.5% SDS, 500 mM NaCl, 50 mM Tris, pH 7.4). The lysate was denatured at 95°C for 5 min and sonicated. Total protein in light and heavy labeled samples was measured by the BCA method and samples were mixed in a 1: 1 protein concentration ratio. One hundred μl of pre-equilibrated streptavidin-sepharose (GE Healthcare, USA) was mixed with the lysate and agitated at room temperature for 1 hour. After extensive washing four times with 1 ml of lysis buffer followed by washing four times with 1 ml of 1 M NaCl, 50 mM Tris (pH 7.4) and 2 × 1 ml of distilled water, the bound proteins were eluted three times with 200 µl 0.5% SDS, 50 mM Tris (pH 6.8), 10% glycerol, 50 mM TCEP at 95°C for 5 minutes. The pooled eluate was concentrated on a 10 kDa cut-off Microcon ultrafiltration column (Merck Millipore, USA) and loaded on the SDS-PAGE gel. After electrophoresis, each gel lane was cut into six slices, the protein reduced and alkylated by iodoacetamide, and digested with trypsin.

Digested peptides were desalted and peptide mixtures were measured using LC-MS consisting of a Dionex UltiMate 3000 RSLCnano system (Thermo Fisher Scientific) coupled via an EASY-spray ion source (Thermo Fisher Scientific) to an Orbitrap Elite mass spectrometer (Thermo Fisher Scientific). Purified peptides were separated on 15 cm EASY-Spray column (75 µm ID, PepMap C18, 2 µm particles, 100 Å pore size; Thermo Fisher Scientific). For each LC-MS/MS analysis, about 1 μg peptides were used for 165 min runs. First 5 min, peptides were loaded onto 2 cm trap column (Acclaim PepMap 100, 100 µm ID, C18, 5 µm particles, 100 Å pore size; Thermo Fisher Scientific) in loading buffer (98.9%/ 1%/0.1%, v/v/v, water/ acetonitrile/ formic acid) at a flow rate of 6 µl/min. Thereafter has been switched valve and peptides were loaded in buffer A (0.1% v/v formic acid in water) and eluted from EASY-Spray column with a linear 120 min gradient of 2% - 35% of buffer B (0.1% v/v formic acid in acetonitrile), followed by a 5 min 90% B wash at a flow rate 300 nl/min. EASY-Spray column temperature was kept at 35°C. Mass spectrometry data were acquired with a Top12 data-dependent MS/MS scan method. Target values for the full scan MS spectra were 1 x 10^6^ charges in the 300-1700 m/z range, with a maximum injection time of 35 ms and resolution of 120,000 at m/z 400. A 2 m/z isolation window and a fixed first mass of 110 m/z was used for MS/MS scans. Fragmentation of precursor ions was performed by CID dissociation with normalized collision energy of 35. MS/MS scans were performed in an ion trap with ion target value of 5 x 10^4^ and maximum injection time of 100 ms. Dynamic exclusion was set to 70 s to avoid repeated sequencing of identical peptides.

MS raw files were analysed by MaxQuant software (version 1.4.1.2), and peptide list were searched against the human Uniprot FASTA database and common contaminants database by the Andromeda search engine with cysteine carbamidomethylation as a fixed modification and N-terminal acetylation, methionine oxidations and thioacyl-lysine as variable modification. The false discovery rate was set to 0.01 for both proteins and peptides with a minimum length of six amino acids and was determined by searching a reverse database. Trypsin was set as protease, and a maximum of two missed cleavages were allowed in the database search. Peptide identification was performed with an allowed initial precursor mass deviation up to 7 ppm and an allowed fragment mass deviation of 0.5 Da. Matching between runs was performed. Quantification of SILAC pairs was performed by MaxQuant with standard setting using a minimum ratio count of 2.

Bioinformatics analyses were performed with the Perseus software.

### Quantitative real time reverse transcription polymerase chain reaction (qRT-PCR)

Total RNA was isolated using RNeasy Mini Kit (Qiagen, MD, USA) according to the manufacturer’s protocol. First strand cDNA was synthesized from 200 ng of total RNA with random hexamer primers using TaqMan Reverse Transcription Reagents (Applied Biosystems). qRT-PCR was performed in ABI Prism 7300 (Applied Biosystems) using SYBR Green I Master Mix (Applied Biosystems).

The following sets of primers were used:

L1CAM: forward: 5’ -CGGCTACTCTGGAGAGGACTAC-3’, reverse: 5’- CGGCACTTGAGTTGAGGAT-3’;

ANT2: forward: 5’-GCCGCCTACTTCGGTATCTATG-3’, reverse: 5’-CAGCAGTGACAGTCTGTGCGAT-3’;

GAPDH: forward: 5’-GCCAAAAGGGTCATCATCTC-3’, reverse: 5’-CTAAGCAGTTGGTGGTGCAG-3’.

The relative quantity of cDNA was estimated by the ΔΔCt method [96]; data were normalized to GAPDH. Samples were measured in triplicates.

### SDS-PAGE and immunoblotting

Cells were washed two times with PBS and then harvested into Laemmli SDS sample lysis buffer, sonicated, and centrifuged at 1600 x g for 10 min. Concentration of proteins was estimated by the BCA method (Pierce Biotechnology Inc., Rockford, USA). Before separation by SDS-PAGE (9% and 14% gels were used), 100 mM DTT and 0.01% bromophenol blue were added to the cell lysates. The same protein amount (40 μg) was loaded into each well. After electrophoresis, proteins were electrotransferred from the gel onto a nitrocellulose membrane using the wet transfer method and detected by specific antibodies combined with horseradish peroxidase-conjugated secondary antibodies (donkey anti-mouse, Bio-Rad, Hercules, CA, USA). Peroxidase activity was detected by ECL (Pierce Biotechnology Inc.). GAPDH or pan-actin were used as a marker of equal loading, as indicated.

### Wound healing assay

A confluent monolayer was disrupted with 10 μl micropipette tip. To remove floating cells, the culture medium was removed and replaced by fresh medium. Recovery of the disrupted area was monitored with time-lapse Leica Microscope DMI6000 for 32 to 48 hours. Cell migration was quantified by comparing the corrupted area with the recovered area according to formula: % of recovering = [(corrupted area – recovered area) / corrupted area] × 100. The size of the area was measured using ImageJ Software; velocity was analyzed with tracking plug-in.

### 3D migration assay

BJ cells sorted according to the L1CAM surface level were serum-starved for 24 hours and then seeded into collagen-containing Matrigel inserted in the column (μ-Slide Chemotaxis 3D, Ibidi, Munich, Germany) according to the manufacturer’s protocol. Migration directed towards serum-containing medium was monitored with time-lapse Leica Microscope DMI6000 for 8 hours. Cell tracking was performed using ImageJ Software. For estimation of velocity and distance of the tracked cells, Chemotaxis and Migration Tool Software (Ibidi) was used. To evaluate random migration, the same procedure was used except that BJ cells were stained with antibody against L1CAM prior to seeding into Matrigel (to discriminate cells according to the L1CAM level based on the intensity of L1CAM surface staining (no fluorescence signal referred to cells with a low L1CAM level; in contrast, high L1CAM cells were those that showed strong fluorescence signal).

### Adhesion assay

A 96-well cell culture plate was coated with laminin-1 (10 μg/ml in Hank's balanced salt solution), fibronectin (20 μg/ml in H_2_O), or rat tail collagen 1 (2 μg/ml in H_2_O) at 37°C at 5% CO_2_ for 1 hour. After washing with 0.1% BSA dissolved in DMEM (WB), the wells were blocked with blocking buffer (0.5% BSA in DMEM) at 37°C at 5% CO_2_ for 1 hour. After subsequent washing with WB, the plate was chilled on ice for 5 minutes, cell suspensions were seeded into the wells and left to adhere in a CO_2_ incubator at 37°C for 30 minutes. Next, the plate was shaken at 200 rpm for 10 to 15 seconds and washed three times with WB. Cells were then fixed with 4% formaldehyde at room temperature for 15 minutes, washed with WB and stained with crystal violet (5 mg/ml in 2% ethanol) for 10 minutes. The plate was subsequently washed several times with H_2_O to get rid of the remains of crystal violet. After drying up the plates, 2% SDS (in H_2_O) was added and incubated at room temperature for 30 minutes. Optical density at 550 nm was estimated by a spectrophotometer (Multiskan EX, Thermo Scientific, USA).

### RNA interference

siRNAs targeting L1CAM (sense: GCAAGAGACAUAUCCACAAtt, antisense: UUGUGGAUAUGUCUCUUGCtg) were introduced into the cells using Lipofectamine™ RNAiMAX (Invitrogen, Carlsbad, CA, USA). Nonsense siRNA sequences (siNT; Ambion, CA, USA) were used as a negative control. shRNAs targeting L1CAM were introduced into the cells using a lentiviral vector. The shRNA vector targeting L1CAM was obtained by insertion of double-stranded oligo: 5'-CCGGCAGCAAGAGACATATCCACAACTCGAGTTGTGGATATGTCTCTTGCTGTTTTT-3' into the *Age*I and *EcoR*I sites in the pLKO.1 vector. A non-target shRNA plasmid was obtained from Sigma (cat. No. SHC016). Lentiviral particles were produced by co-transfection with pMD2.G and psPAX2 (a kind gift from Didier Trono) in HEK293T cells as described previously [93]. Stably transduced cells were selected after 7 days by growing in media containing 2.5 µg/ml of puromycin.

### RhoA activity measurement

RhoA activity was measured using G-LISA Activation Assay (Cytoskeleton, Inc). The procedure was performed according to manufacturer’s protocol.

### Determination of cell nuclei polarization

Confluent cell monolayer was scratched by pipette tip. After 4 hours, cells were stained (live) with the L1CAM antibody, then fixed, permeabilized and stained with antibody against a marker of MTOC, γ-tubulin. For determination of cell polarization, the position of MTOC was measured relatively to perpendicular lines (90°) positioned toward the scratch line [97]. The nuclei with MTOC present within the perpendicular lines were considered as polarized.

## Supplementary Material

Supplementary File

Supplementary Table 1

Supplementary Video 1

Supplementary Video 2
