# Supplementary material for: Induction, regulation and roles of neural adhesion molecule L1CAM in cellular senescence
Source: Aging (Albany NY). 2018 Mar 28;10(3):434–62. doi: 10.18632/aging.101404 (PMC5892697; doi:10.18632/aging.101404)
Supplement: Supplementary File [file aging-10-101404-s001.pdf]

# SUPPLEMENTARY MATERIAL

Please browse the Full Text version to see:

**Supplementary Table 1.** List of proteins with significantly changed expression identified by mass spectrometry.

**Supplementary Video 1.** Wound healing assay of proliferating BJ fibroblasts.

**Supplementary Video 2.** Wound healing assay of replicatively senescent BJ fibroblasts.

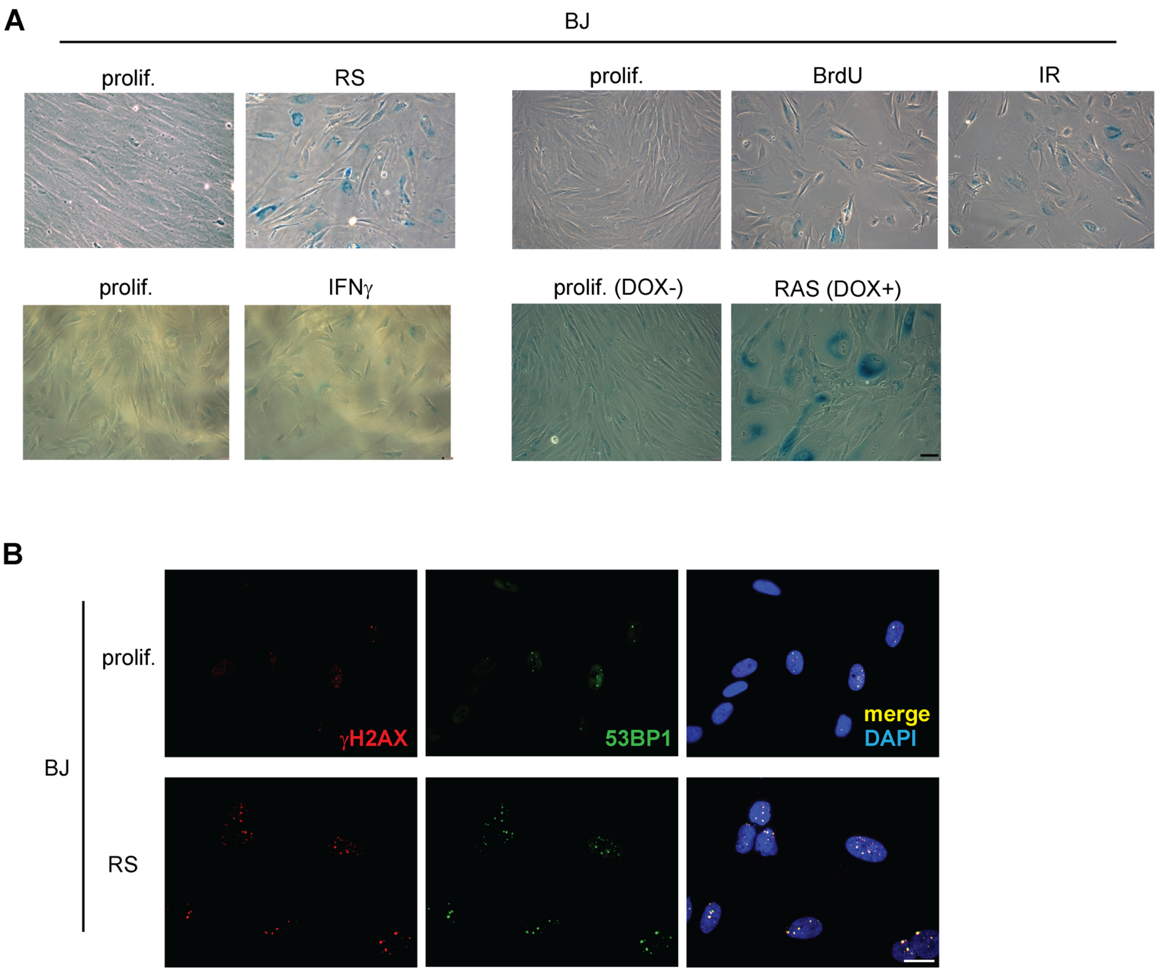


**Supplementary Figure 1.** (**A**) Senescence-associated β-galactosidase staining of BJ fibroblasts brought to senescence by various means: replicatively senescent (by splitting cell culture in ratio 1 : 2 until proliferation exhaustion at PD 85), by treatment of BJ cells (PD 32) with 100 μM BrdU, by irradiation of BJ cells (PD 32; IR, 20 Gy), by exposure of BJ cells (PD 35) to IFNγ (500 U/ml; for 21 days) and by tetracycline-inducible oncogenic H-RAS (see Materials and Methods and reference [94]). (**B**) DNA damage response of proliferating (PD 30) and replicatively senescent (PD 85) BJ fibroblasts detected as DNA damage foci with antibodies against γH2AX and 53BP1. Scale bar, 20 μm.

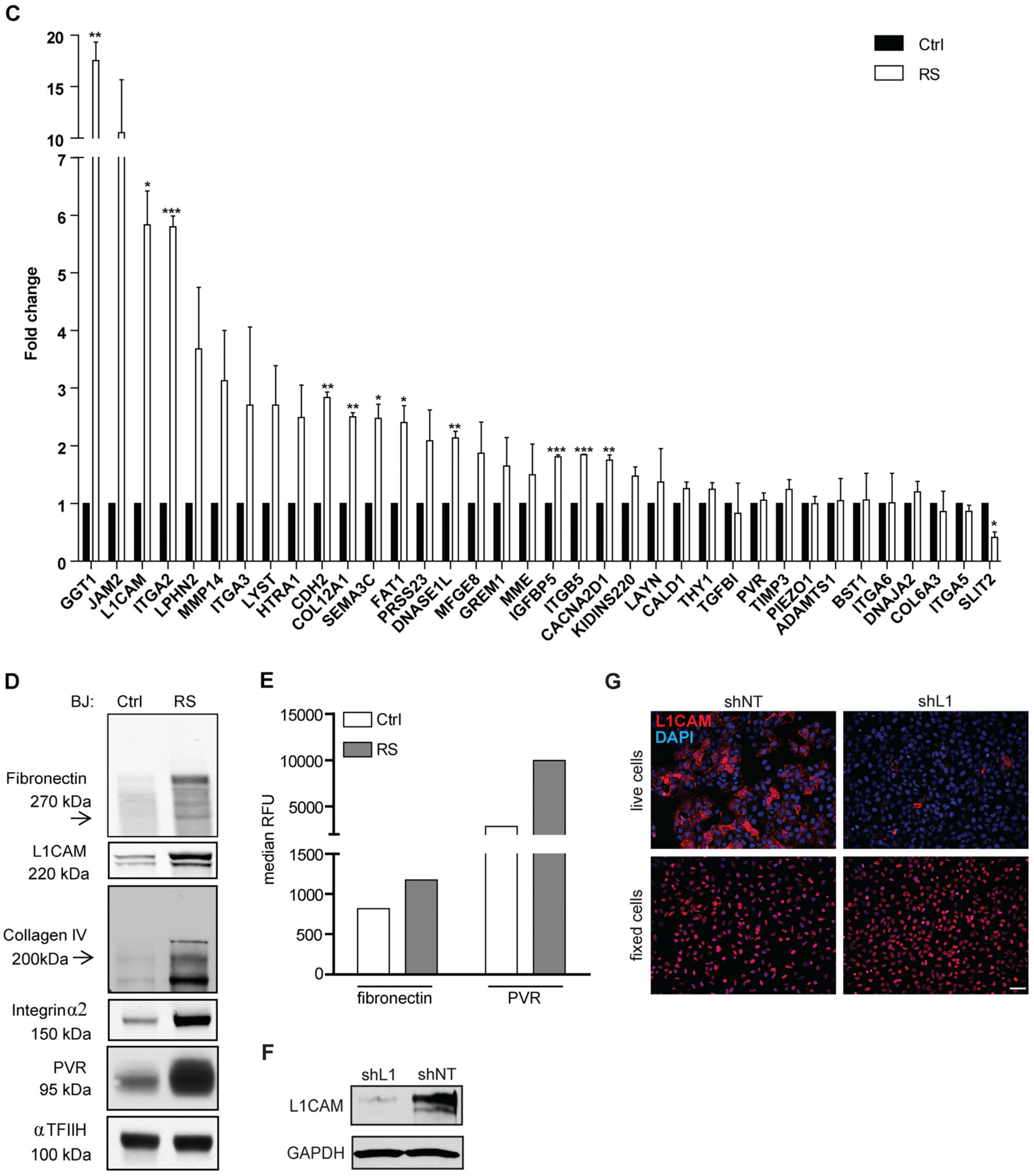


**Supplementary Figure 1**. (**C**) Transcript levels estimated by real time quantitative RT-PCR in subset of most upregulated hits obtained by mass spectrometry analysis in proliferating (Ctrl) and replicatively senescent (RS) BJ fibroblasts. GAPDH was used as a reference gene. Measurements were performed in two independent replicates. $p < 0.05$ (*); $p < 0.01$ (**); $p < 0.001$ (***), (**D**) Immunoblot comparing total protein levels of fibronectin, L1CAM, collagen IV, integrin $\alpha$2 and PVR in proliferating (Ctrl) and replicatively senescent (RS) BJ fibroblasts. αTFIIH was used as a loading control. (**E**) FACS analysis of surface expression of fibronectin and PVR in proliferating (Ctrl) and replicatively senescent (RS) BJ cells. (**F**) Effectiveness of shRNA-mediated downregulation of L1CAM using lentiviral transduction (shL1) compared to control U2OS cells transduced with non-targeting shRNA (shNT) detected by Western blotting. GAPDH was used as a loading control. (**G**) Immunofluorescence staining of live control (shNT) and shL1CAM-treated U2OS cells (shL1) with L1CAM antibody (upper row) and after cell permeabilization (lower row; note that the signal in permeabilized cells is nonspecific). Scale bars, 50 µm.

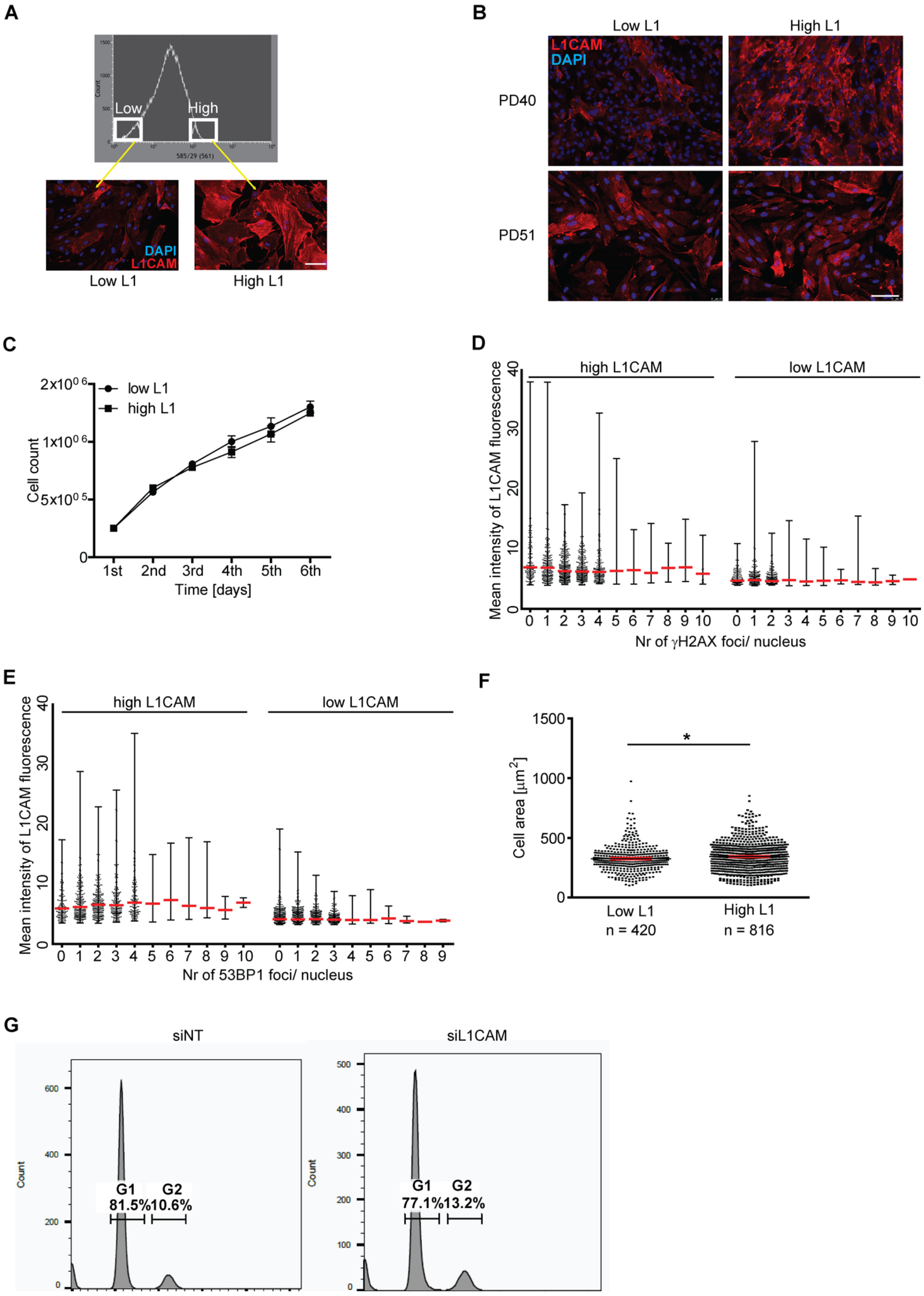


**Supplementary Figure 2.** (**A**) Fluorescence-associated cell sorting of replicatively senescent BJ fibroblasts. Live cells were sorted according to their surface L1CAM level into two subpopulations with low L1CAM and high L1CAM. Scale bar, 50 µm. (**B**) Immunofluorescence staining of surface L1CAM in sorted BJ fibroblasts (PD 40) and after additional 11 population doublings (PD 51). Scale bar, 50 µm. (**C**) Comparison of proliferation of L1CAM 'high' and 'low' BJ cells sorted by FACS. Quantification of number of γH2A.X (**D**) and 53BP1 (**E**) DNA damage foci per cell nucleus plotted relative to the L1CAM surface fluorescent intensity. Replicatively senescent BJ fibroblasts were presorted according to L1CAM surface level by FACS (L1CAM$^{high}$ versus L1CAM$^{low}$). (**F**) Cell area of BJ fibroblasts sorted for L1CAM high and low level. (**G**) FACS analysis of DNA content in replicatively BJ fibroblasts after knockdown of L1CAM (right) using propidium iodide staining. Control cells were transfected with non-specific siRNA (siNT; left).

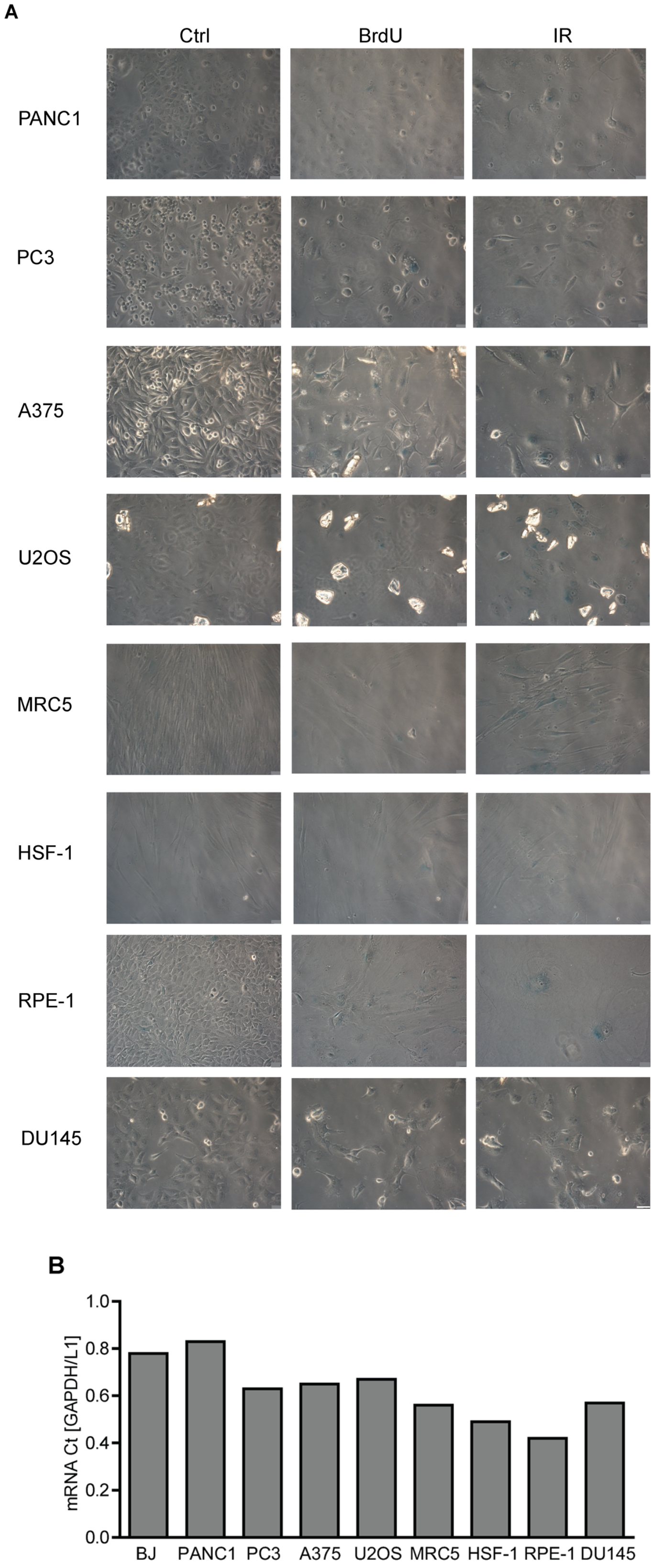


**Supplementary Figure 3**. (**A**) Senescence-associated β-galactosidase staining of PANC-1, PC3, A375, U2OS, MRC5, HSF-1, RPE-1 and DU145 cells brought to premature senescence by BrdU and IR (10 Gy). Scale bar, 50 µm. (**B**) Comparison of basal L1CAM transcript levels in different cell types calculated as an average GAPDH to L1CAM Ct values ratio.

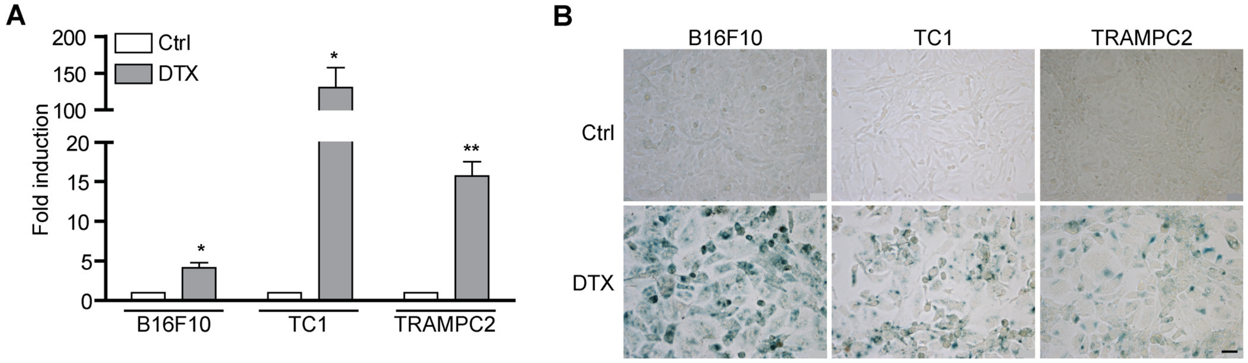


**Supplementary Figure 4**. L1CAM transcript levels (**A**) and senescence-associated β-galactosidase activity (**B**) estimated in mouse cell lines B16F10, TC1, and TRAMPC2 after exposure to docetaxel (0.75 μM, 4 days). Scale bar, 50 μm.

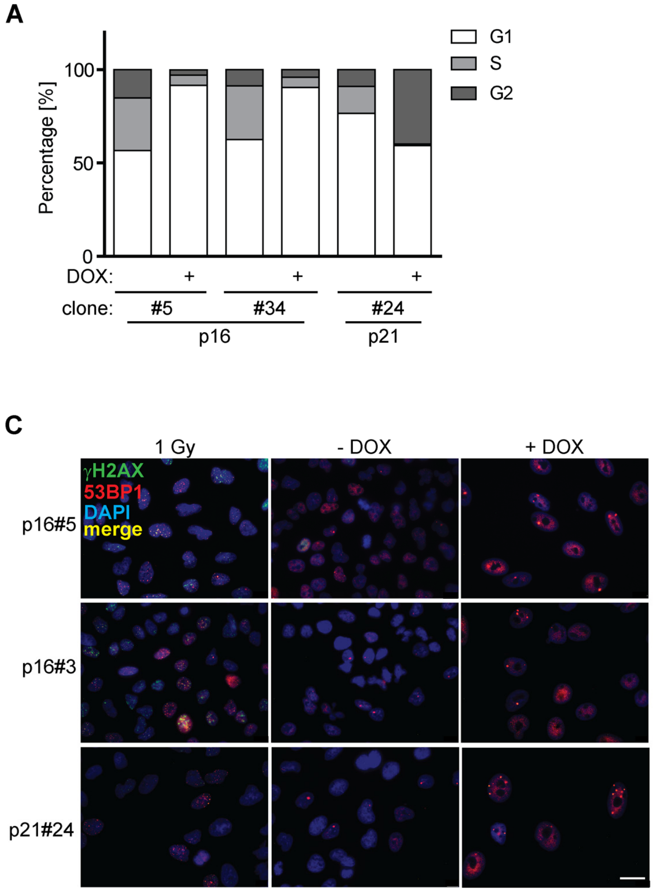


**Supplementary Figure 5**. (**A**) Cell cycle arrest after overexpression of cyclin-dependent kinase inhibitors p16 and p21 (induction with doxycycline, DOX+). (**B**) Senescence-associated β-galactosidase staining of H28 clones after cell cycle arrest (DOX+). (**C**) DNA damage markers (γH2AX and 53BP1) staining. Cells irradiated with dose of 1Gy were used as a staining control (40 minutes after irradiation). Scale bars, 20 μm.

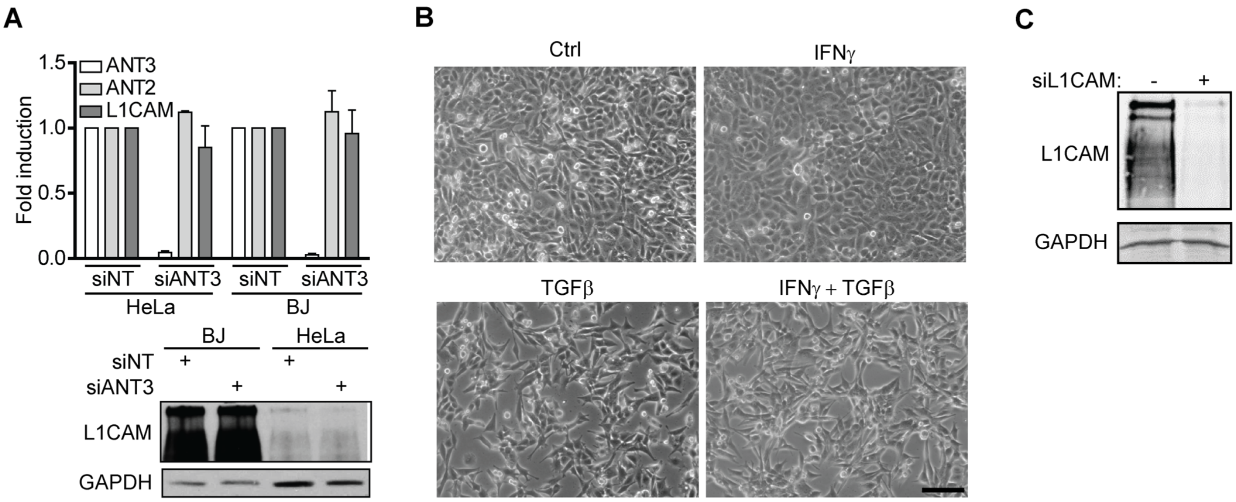


**Supplementary Figure 6**. (**A**) mRNA of ANT3, ANT2, and L1CAM after downregulation of ANT3 using RNA interference in HeLa and BJ cells (upper chart) and total L1CAM protein in Hela and BJ cells after downregulation of ANT3 using RNA interference (lower panel). GAPDH was used as a loading control. (**B**) Phase contrast microscopic images of A375 cells after exposure to 500 U/ml IFNγ, 10 ng/ml TGFβ, or their combination for 4 days. (**C**) Effectiveness of L1CAM downregulation by L1CAM siRNA in replicatively senescent BJ fibroblasts detected by Western blotting (siNT, control; L1CAM). GAPDH was used as a loading control. Scale bar, 100 µm.

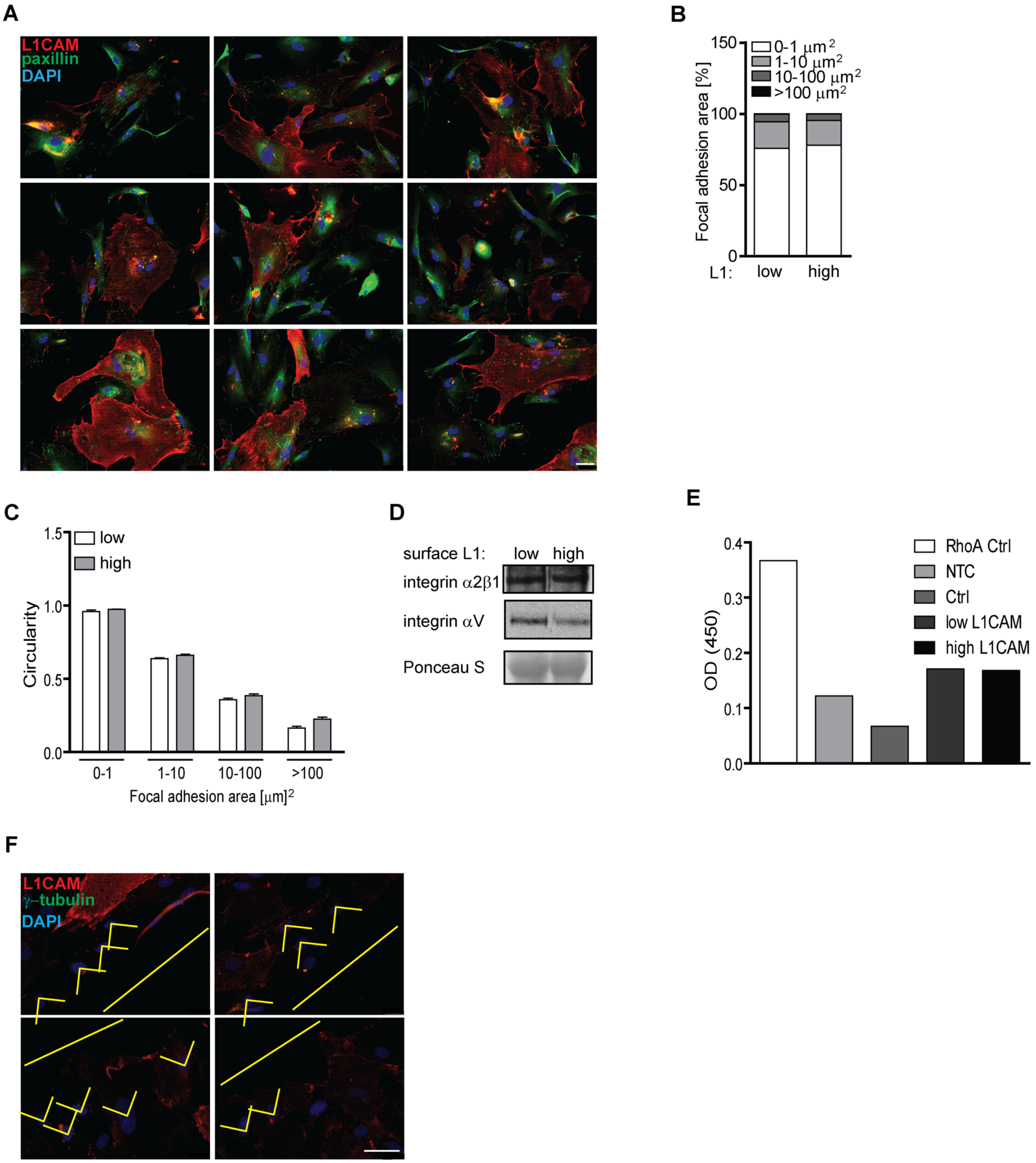


**Supplementary Figure 7.** (**A**) Fluorescence staining of L1CAM (surface labeling) and paxillin (after permeabilization) in non-sorted replicatively senescent BJ fibroblasts. (**B**) The ratio of adhesion (paxillin) numbers and area in low L1CAM and high L1CAM BJ replicatively senescent cells. (**C**) Circularity of adhesions in individual area categories. (**D**) Western blot detecting integrins in low and high L1CAM cells (BJ replicatively senescent fibroblasts). β–actin was used as a loading control. (**E**) RhoA activity in low and high L1CAM cells. "RhoA control" was part of the kit compounds; as negative control (NTC) were used cells with overnight serum starvation and control cells (Ctrl) were cells treated with 2 μM lysophosphatidic acid. (**F)** Polarization of nuclei after wound healing. Yellow line represents the direction of scratch ("wound) in cells monolayer. "v"-shaped perpendicular yellow lines for determination of nuclei polarization. Scale bar, 50 μm. All experiments were performed in three independent replicates. For statistics, two-tailed Student's t-test was used.
