# Supplementary material for: Induction, regulation and roles of neural adhesion molecule L1CAM in cellular senescence
Source: Aging (Albany NY). 2018 Mar 28;10(3):434–62. doi: 10.18632/aging.101404 (PMC5892697; doi:10.18632/aging.101404)
Supplement: Supplementary Table 1 [file aging-10-101404-s002.docx]

**Supplementary Table 1.** The list of proteins with significantly changed expression identified by mass spectrometry. Listed proteins are the result of comparison of proliferating (normal, “N”) and replicatively senescent (“S”) BJ fibroblasts.

| Average LogFC  (N/S) | Average LogFC  (S/N) | Intensity | Test statistic | Protein IDs | Majority protein IDs | Protein names | Gene names |
| --- | --- | --- | --- | --- | --- | --- | --- |
| -2.635226 | 3.371001 | 7.360006132 | -23.73301783 | P36268-2;  B5MCK8;  P36268;  P19440;  P36268-3;  E7ET76;  A6NGU5;  P19440-2;  E7ETR7;  Q14390;  B5MC36;  P19440-3;  B5MD39;  J3KR28;  B5MC34 | P36268-2;  B5MCK8;  P36268;  P19440;  P36268-3;  E7ET76;  A6NGU5;  P19440-2 | Gamma-glutamyltranspeptidase 2;  Gamma-glutamyltranspeptidase 2 heavy chain;  Gamma-glutamyltranspeptidase 2 light chain;  Gamma-glutamyltranspeptidase 1;  Gamma-glutamyltranspeptidase 1 heavy chain;  Gamma-glutamyltranspeptidase 1 light chain;  Putative gamma-glutamyltranspeptidase 3;  Putative gamma-glutamyltranspeptidase 3 heavy chain;  Putative gamma-glutamyltranspeptidase 3 light chain | GGT2;  GGT1;  GGT3P |
| -1.905034 | 2.866394 | 7.451433332 | -27.75607925 | P32004-3;  P32004-2;  F5H1H0;  P32004;  F5H025;  H0Y5C3;  H3BLW5;  E9PHJ4;  E7EMY4;  E7EVM4;  E7EPI4 | P32004-3;  P32004-2;  F5H1H0;  P32004;  F5H025 | Neural cell adhesion molecule L1 | L1CAM |
| -0.8198013 | 2.563353 | 8.94063102 | -13.49351502 | E9PIM6;  P04216;  E9PNQ8;  J3QRJ3 | E9PIM6;  P04216;  E9PNQ8;  J3QRJ3 | Thy-1 membrane glycoprotein | THY1 |
| -0.6515636 | 2.280897 | 8.187012807 | -3.314451 | Q6NZI2;  Q6NZI2-2;  Q6NZI2-3 | Q6NZI2;  Q6NZI2-2 | Polymerase I and transcript release factor | PTRF |
| -1.056221 | 2.215119 | 7.597574532 | -29.24840775 | Q10588;  A6NC48;  H0Y984;  H0Y9Q9;  H0Y8G4 | Q10588;  A6NC48;  H0Y984 | ADP-ribosyl cyclase 2 | BST1 |
| -1.125573 | 2.088058 | 7.692547303 | -14.07066679 | D6RGG3;  Q99715;  Q99715-4;  Q99715-2;  H0Y4P7;  H0Y5N9;  H0Y991 | D6RGG3;  Q99715;  Q99715-4;  Q99715-2 | Collagen alpha-1(XII) chain | COL12A1 |
| -2.187379 | 2.041699 | 6.935658386 | -19.17918278 | Q9UHI8;  E5RJR7;  H7C206;  E5RI60 | Q9UHI8 | A disintegrin and metalloproteinase with thrombospondin motifs 1 | ADAMTS1 |
| -0.9195065 | 1.90616 | 7.760331996 | -18.15224672 | Q05682-5;  E9PGZ1;  Q05682-4;  F5H1Z9;  E7EX44;  Q05682-3;  Q05682-6;  Q05682-2;  Q05682;  C9J813;  C9JEK3 | Q05682-5;  E9PGZ1;  Q05682-4;  F5H1Z9;  E7EX44;  Q05682-3;  Q05682-6;  Q05682-2;  Q05682;  C9J813 | Caldesmon | CALD1 |
| -1.562218 | 1.905389 | 8.28831631 | -23.64976861 | P26006;  P26006-1;  D6R9X8;  H0YA49;  B4DDT0 | P26006;  P26006-1 | Integrin alpha-3;  Integrin alpha-3 heavy chain;  Integrin alpha-3 light chain | ITGA3 |
| -2.615552 | 1.863978 | 8.272259443 | -7.367754393 | P35625 | P35625 | Metalloproteinase inhibitor 3 | TIMP3 |
| -0.7482576 | 1.856946 | 7.015443588 | -14.01638604 | Q92508;  E7EUT2;  H7C2J5;  H0YB49;  H3BRR7 | Q92508 | Piezo-type mechanosensitive ion channel component 1 | PIEZO1 |
| -0.8403708 | 1.824809 | 7.734151503 | -19.7432219 | P54289-5;  P54289-2;  P54289 | P54289-5;  P54289-2;  P54289 | Voltage-dependent calcium channel subunit alpha-2/delta-1;  Voltage-dependent calcium channel subunit alpha-2-1;  Voltage-dependent calcium channel subunit delta-1 | CACNA2D1 |
| -0.9346483 | 1.818605 | 7.536381659 | -11.5355114 | P49184;  A6QRJ0;  Q5HY40;  E7ESG1;  E7EUJ0 | P49184;  A6QRJ0 | Deoxyribonuclease-1-like 1 | DNASE1L1 |
| -0.9806778 | 1.676899 | 6.925193444 | -9.263101219 | O60565;  O60565-2 | O60565;  O60565-2 | Gremlin-1 | GREM1 |
| -0.4855596 | 1.659285 | 8.030559245 | -9.665333575 | P23229-4;  P23229-2;  P23229-5;  P23229-3;  P23229-9;  P23229-6;  P23229;  P23229-7;  H7BZ97;  C9JXX7 | P23229-4;  P23229-2;  P23229-5;  P23229-3;  P23229-9;  P23229-6;  P23229;  P23229-7 | Integrin alpha-6;  Integrin alpha-6 heavy chain;  Integrin alpha-6 light chain | ITGA6 |
| -1.806288 | 1.640436 | 7.018159374 | -6.140773349 | P29279;  P29279-2 | P29279;  P29279-2 | Connective tissue growth factor | CTGF |
| 0.5815923 | 1.49904 | 6.478061208 | -2.912109038 | Q76M96;  Q76M96-2;  H7C5K4;  C9J8I6 | Q76M96;  Q76M96-2;  H7C5K4 | Coiled-coil domain-containing protein 80 | CCDC80 |
| -0.09137043 | 1.469535 | 7.021313365 | -12.18252902 | P56199 | P56199 | Integrin alpha-1 | ITGA1 |
| -1.136728 | 1.368992 | 7.048868896 | -20.42575459 | Q14517;  D6RCE4;  H0Y9C8;  H0Y9H4 | Q14517 | Protocadherin Fat 1;  Protocadherin Fat 1, nuclear form | FAT1 |
| 0.50196 | 1.348232 | 6.428037553 | -3.791468659 | E7EPS8;  P28827;  P28827-2;  F8WA38;  E7EVX9;  J3KRV2;  B1AJS0;  B1AJR6;  O14522;  B1AJR8;  B1AJR9;  O14522-1 | E7EPS8;  P28827;  P28827-2;  F8WA38;  E7EVX9 | Receptor-type tyrosine-protein phosphatase mu | PTPRM |
| -1.369706 | 1.306846 | 7.900623806 | -15.79799287 | A8MWK3;  P19022;  C9J126;  C9J8J8;  P55283-2;  P55283 | A8MWK3;  P19022 | Cadherin-2 | CDH2 |
| -0.8834575 | 1.278044 | 8.015988105 | -10.87469123 | P35580;  P35580-2;  F8W6L6;  P35580-3;  P35580-4;  E7ERA5 | P35580;  P35580-2;  F8W6L6;  P35580-3;  P35580-4 | Myosin-10 | MYH10 |
| -0.4353584 | 1.255742 | 8.022881813 | -55.53520178 | Q9UBG0;  E7EME3 | Q9UBG0 | C-type mannose receptor 2 | MRC2 |
| -0.1753502 | 1.232292 | 7.71603675 | -18.27778286 | Q15746-2;  Q15746-4 | Q15746-2;  Q15746-4 | Myosin light chain kinase, smooth muscle;Myosin light chain kinase, smooth muscle, deglutamylated form | MYLK |
| -0.2771412 | 1.222618 | 6.750824551 | -6.199272727 | E9PNW4;  P13987;  E9PR17;  H0YET2 | E9PNW4;  P13987;  E9PR17;  H0YET2 | CD59 glycoprotein | CD59 |
| -0.340934 | 1.16221 | 7.656126962 | -11.40986537 | P22413;  E9PE72 | P22413 | Ectonucleotide pyrophosphatase/phosphodiesterase family member 1;  Alkaline phosphodiesterase I;  Nucleotide pyrophosphatase | ENPP1 |
| -0.481626 | 1.157108 | 7.849873742 | -11.85777316 | P35052;  H7C410;  H7C024;  C9J4Y6;  H7BZE9;  H7BZL4 | P35052;  H7C410;  H7C024 | Glypican-1;  Secreted glypican-1 | GPC1 |
| -0.5312016 | 1.13829 | 7.537655417 | -4.398699388 | P54652 | P54652 | Heat shock-related 70 kDa protein 2 | HSPA2 |
| -1.252604 | 1.124189 | 7.260858132 | -8.212856566 | P15151-3;  P15151-2;  P15151-4;  P15151 | P15151-3;  P15151-2;  P15151-4;  P15151 | Poliovirus receptor | PVR |
| -0.2930318 | 1.081544 | 7.950447616 | -4.531682786 | Q09666;  E9PQE3;  E9PJZ0;  E9PKR9;  E9PJC6;  E9PLK4;  Q09666-2 | Q09666 | Neuroblast differentiation-associated protein AHNAK | AHNAK |
| -0.2698426 | 1.010279 | 7.123198075 | -10.38509764 | E9PMR4;  P48509 | E9PMR4;  P48509 | CD151 antigen | CD151 |
| -0.8762278 | 1.003026 | 8.740236331 | -7.878293555 | P07355;  P07355-2;  H0YN42;  H0YMD0;  H0YMU9;  A6NMY6;  H0YKS4;  H0YM50;  H0YNP5;  H0YMM1;  H0YKZ7;  H0YLV6;  H0YMT9;  H0YKX9;  H0YN28;  H0YL33;  H0YKL9;  H0YMW4;  H0YKV8;  H0YMD9;  H0YNA0;  H0YNB8;  H0YN52;  H0YKN4;  H0YLE2 | P07355;  P07355-2;  H0YN42;  H0YMD0;  H0YMU9;  A6NMY6;  H0YKS4;  H0YM50;  H0YNP5;  H0YMM1 | Annexin A2;  Annexin;  Putative annexin A2-like protein | ANXA2;  ANXA2P2 |
| -1.129987 | 1.00101 | 9.088667553 | -5.424561495 | P08670;  B0YJC4;  B0YJC5;  Q5JVS8;  H7C5W5;  P17661;  P41219;  P41219-2;  F8W835;  P07197-2;  P07196;  E7EMV2;  E7ESP9;  P07197;  P12036-2;  P12036 | P08670;  B0YJC4;  B0YJC5 | Vimentin | VIM |
| -0.86436 | 0.9668734 | 7.94019762 | -12.91442733 | Q08431-2;  F5GZN3;  F5H7N9;  Q08431;  X6R3G6;  Q08431-3;  H0YKS8 | Q08431-2;  F5GZN3;  F5H7N9;  Q08431;  X6R3G6;  Q08431-3;  H0YKS8 | Lactadherin;  Lactadherin short form;Medin | MFGE8 |
| -0.4771206 | 0.9334971 | 7.771205192 | -8.84920598 | J3QQV2;  J3QSC3;  O15427;  J3QQS9;  J3KTI8;  J3KT83;  J3QRA0;  J3QLE3;  J3KTM6;  J3QRU2 | J3QQV2;  J3QSC3;  O15427;  J3QQS9;  J3KTI8;  J3KT83;  J3QRA0 | Monocarboxylate transporter 4 | SLC16A3 |
| -0.5684305 | 0.8954578 | 7.284475853 | -4.242339781 | P05121;  P05121-2 | P05121;  P05121-2 | Plasminogen activator inhibitor 1 | SERPINE1 |
| -0.2510408 | 0.8626325 | 7.199919652 | -9.841252103 | Q16585;  B7Z635;  H0YA15;  H0Y8J3 | Q16585;  B7Z635 | Beta-sarcoglycan | SGCB |
| -0.08886836 | 0.8542351 | 8.206313052 | -6.399063413 | Q6UVY6;  Q6UVY6-2;  A6PVS1 | Q6UVY6;  Q6UVY6-2 | DBH-like monooxygenase protein 1 | MOXD1 |
| -0.541765 | 0.8422145 | 8.052578525 | -2.94282963 | P02545;  P02545-3;  P02545-5;  P02545-4;  P02545-6;  H0YAB0 | P02545;  P02545-3;  P02545-5;  P02545-4;  P02545-6 | Prelamin-A/C;  Lamin-A/C | LMNA |
| -1.015987 | 0.8093321 | 7.658593248 | -7.175469028 | Q92743;  H0Y7G9 | Q92743;  H0Y7G9 | Serine protease HTRA1 | HTRA1 |
| -0.1059855 | 0.8074374 | 6.385588401 | -3.94627221 | P17948-2;  P17948-3;  P17948;  P17948-4 | P17948-2;  P17948-3;  P17948;  P17948-4 | Vascular endothelial growth factor receptor 1 | FLT1 |
| -0.3820621 | 0.7911056 | 7.729359206 | -5.020286273 | Q9NR99 | Q9NR99 | Matrix-remodeling-associated protein 5 | MXRA5 |
| -0.5018922 | 0.7211539 | 8.16181704 | -9.369371771 | P05362;  E7ESS4;  K7EKL8 | P05362;  E7ESS4 | Intercellular adhesion molecule 1 | ICAM1 |
| -0.8823935 | 0.7083197 | 7.16521464 | -5.336774591 | B7Z1R5;  P38606;  C9JA17;  C9JVW8 | B7Z1R5;  P38606 | V-type proton ATPase catalytic subunit A | ATP6V1A |
| -0.1833921 | 0.6391395 | 8.367691298 | -3.712491978 | P12814 | P12814 | Alpha-actinin-1 | ACTN1 |
| -0.5411816 | 0.6373784 | 6.342225229 | -12.45568423 | Q9UP95-3;  Q9UP95-5;  Q9UP95-2;  Q9UP95-6;  Q9UP95;  I3L4N6;  Q9UP95-7;  Q9UP95-4;  Q9H2X9-2;  Q9H2X9 | Q9UP95-3;  Q9UP95-5;  Q9UP95-2;  Q9UP95-6;  Q9UP95;  I3L4N6;  Q9UP95-7;  Q9UP95-4 | Solute carrier family 12 member 4 | SLC12A4 |
| 1.318747 | 0.626112 | 6.55544513 | 3.226086374 | Q9P2B2 | Q9P2B2 | Prostaglandin F2 receptor negative regulator | PTGFRN |
| -1.383362 | 0.6195538 | 6.439758883 | -8.390679352 | P50443 | P50443 | Sulfate transporter | SLC26A2 |
| -0.4099873 | 0.6084308 | 8.200850498 | -11.03257944 | F5H6E2;  O00159-3;  O00159;  O00159-2;  I3L501;  I3L204;  I3L4D4;  I3L3Y6;  I3L168;  I3L3F5 | F5H6E2;  O00159-3;  O00159;  O00159-2 | Unconventional myosin-Ic | MYO1C |
| 1.119024 | 0.5490283 | 8.204092838 | 7.417548163 | Q12884;  B4DLR2;  Q12884-2;  C9J131;  H7C4D9;  H0YG61;  F8WF29 | Q12884;  B4DLR2 | Seprase | FAP |
| -0.5252888 | 0.5367492 | 8.712961003 | -8.776629671 | P35613-3;  P35613-4;  P35613-2;  P35613;  4GN83;  R4GMX5;  I3L192 | P35613-3;  P35613-4;  P35613-2;  P35613;  R4GN83;  R4GMX5;  I3L192 | Basigin | BSG |
| -0.1385051 | 0.5366498 | 7.509766873 | -4.370782114 | O43707;  O43707-2;  O43707-3;  F5GXS2;  H7C144;  K7EJH8;  K7EP19;  D6RH00 | O43707;  O43707-2;  O43707-3;  F5GXS2 | Alpha-actinin-4 | ACTN4 |
| -0.7124568 | 0.5221562 | 7.141198845 | -6.219432475 | P29317;  Q9UF33-2;  J3KR66;  Q9UF33-3;  B7Z6Q8;  B1AKC9;  E9PG71;  P54764;  P29323-2;  P29323-3;  Q15375-2;  Q15375-4;  Q15375;  Q9UF33;  P29323 | P29317 | Ephrin type-A receptor 2 | EPHA2 |
| -0.2597891 | 0.5181323 | 8.35328134 | -8.73467869 | Q6YHK3;  Q6YHK3-4;  Q6YHK3-2;  Q6YHK3-3 | Q6YHK3;  Q6YHK3-4;  Q6YHK3-2 | CD109 antigen | CD109 |
| -0.3001286 | 0.514097 | 8.779177866 | -2.963840615 | Q15149-4;  Q15149-7;  Q15149-8;  Q15149-9;  Q15149-5;  Q15149-6;  Q15149-2;  Q15149;  E9PMV1;  E9PKG0;  E9PIA2;  E9PPU0;  E9PQ28;  H0YDN1 | Q15149-4;  Q15149-7;  Q15149-8;  Q15149-9;  Q15149-5;  Q15149-6;  Q15149-2;  Q15149 | Plectin | PLEC |
| -0.2712871 | 0.5065004 | 7.892784471 | -5.3323466 | Q9Y639-3;  Q9Y639-1;  Q9UFM8;  Q9Y639-4;  H7BXJ1;  Q9Y639-5;  F5GZD0;  Q9Y639;  H3BQ94;  H3BU51;  F5GYM7 | Q9Y639-3;  Q9Y639-1;  Q9UFM8;  Q9Y639-4;  H7BXJ1;  Q9Y639-5;  F5GZD0;  Q9Y639 | Neuroplastin | NPTN;  DKFZp566H1924 |
| -0.2973957 | 0.4972291 | 7.410726633 | -4.017317334 | O75326-2;  O75326;  F5GYX3;  H3BMF9 | O75326-2;  O75326;  F5GYX3 | Semaphorin-7A | SEMA7A |
| -0.2550467 | 0.4927248 | 8.287420996 | -6.303314899 | P08195-2;  P08195-3;  F5GZS6;  P08195;  J3KPF3;  P08195-4;  H0YFS2;  F5H0E2;  H0YFX4;  F5GZI0;  F5H867 | P08195-2;  P08195-3;  F5GZS6;  P08195;  J3KPF3;  P08195-4 | 4F2 cell-surface antigen heavy chain | SLC3A2 |
| -0.822503 | 0.4302852 | 8.722609246 | -12.72046596 | P05556;  P05556-2;  P05556-5;  P05556-4;  P05556-3;  E7EQW5;  H7C4K3;  C9JPK5;  Q5T3E6;  E9PLR6;  E7EUI6 | P05556;  P05556-2;  P05556-5;  P05556-4;  P05556-3 | Integrin beta-1 | ITGB1 |
| -1.067281 | 0.4262111 | 7.12765542 | -7.69272399 | E9PGC8;  P78559;  P78559-2;  J3KPX8 | E9PGC8;  P78559;  P78559-2;  J3KPX8 | Microtubule-associated protein 1A;  MAP1A heavy chain;  MAP1 light chain LC2 | MAP1A |
| -1.253223 | 0.4208328 | 7.025223915 | -7.570945305 | P11498;  E9PS68;  E9PRE7;  B4DN00 | P11498 | Pyruvate carboxylase, mitochondrial | PC |
| -0.9725628 | 0.4196467 | 8.417438476 | -7.162131575 | P04083;  Q5T3N1;  Q5T3N0 | P04083;  Q5T3N1 | Annexin A1 | ANXA1 |
| -0.3668757 | 0.4032677 | 7.774436669 | -5.282372492 | P05023-3;  P05023-4;  P05023;  P05023-2;  M0R116;  P13637;  P13637-2;  P13637-3;  B1AKY9;  P50993;  H0Y7C1;  Q13733;  Q5TC01;  Q5TC02;  E9PRA5;  M0QXF2;  P20648;  P54707;  P54707-2 | P05023-3;  P05023-4;  P05023;  P05023-2 | Sodium/potassium-transporting ATPase subunit alpha-1 | ATP1A1 |
| -1.317355 | 0.3936349 | 6.708556861 | -4.450898052 | B7Z588;  O14763-2;  O14763 | B7Z588;  O14763-2;  O14763 | Tumor necrosis factor receptor superfamily member 10B | TNFRSF10B |
| 1.104494 | 0.3890782 | 5.965794158 | 9.421417071 | Q9HA72-3;  Q9HA72-2;  Q9HA72 | Q9HA72-3;  Q9HA72-2;  Q9HA72 | Calcium homeostasis modulator protein 2 | CALHM2 |
| -0.9797432 | 0.3855966 | 6.713658516 | -2.581286037 | Q66K79-3;  Q66K79-2;  Q66K79;  E7EQK1 | Q66K79-3;  Q66K79-2;  Q66K79;  E7EQK1 | Carboxypeptidase Z | CPZ |
| 0.6715652 | 0.3720605 | 7.898851284 | 3.534624904 | P17693;  Q5RJ85 | P17693;  Q5RJ85 | HLA class I histocompatibility antigen, alpha chain G | HLA-G |
| -0.1316925 | 0.3637879 | 8.261667229 | -5.382906415 | Q6EMK4 | Q6EMK4 | Vasorin | VASN |
| -0.3485005 | 0.3373114 | 8.60963709 | -4.320996322 | Q14315-2;  Q14315 | Q14315-2;  Q14315 | Filamin-C | FLNC |
| -0.275472 | 0.3362834 | 9.855234378 | -9.498885687 | P63261;  I3L3I0;  I3L1U9;  I3L4N8;  K7EM38;  I3L3R2;  J3KT65;  C9JUM1;  Q6S8J3;  Q9BYX7;  P0CG38;  P0CG39 | P63261;  I3L3I0;  I3L1U9;  I3L4N8 | Actin, cytoplasmic 2;  Actin, cytoplasmic 2, N-terminally processed | ACTG1 |
| -1.778828 | 0.3220435 | 7.284137345 | -14.29312658 | Q8TF66;  Q8TF66-2 | Q8TF66;  Q8TF66-2 | Leucine-rich repeat-containing protein 15 | LRRC15 |
| -0.6020656 | 0.3130136 | 8.089905111 | -6.812376232 | P04156-2;  A2A2V1;  P04156;  X6RKS3 | P04156-2;  A2A2V1;  P04156;  X6RKS3 | Major prion protein | PRNP |
| 0.6635723 | 0.2973675 | 8.57623766 | 3.737426432 | P30479;  P30481 | P30479;  P30481 | HLA class I histocompatibility antigen, B-41 alpha chain;HLA class I histocompatibility antigen, B-44 alpha chain | HLA-B |
| -0.6688223 | 0.2720232 | 7.491893729 | -25.21062698 | P04899;  P04899-4;  P04899-6;  P04899-3;  P04899-5;  P04899-2;  F5GZL8 | P04899;  P04899-4;  P04899-6;  P04899-3;  P04899-5;  P04899-2;  F5GZL8 | Guanine nucleotide-binding protein G(i) subunit alpha-2 | GNAI2 |
| -0.3451979 | 0.2651968 | 7.825860844 | -3.066141086 | P09525;  Q6P452;  B4DDF9 | P09525;  Q6P452;  B4DDF9 | Annexin A4;  Annexin | ANXA4 |
| -0.9221008 | 0.2239167 | 7.939873532 | -3.89511965 | P07237;  H7BZ94;  F5H8J2;  I3L398;  I3L312;  I3L4M2;  H0Y3Z3;  I3NI03;  I3L3U6;  I3L3P5;  I3L1Y5;  I3L514;  I3L0S0 | P07237;  H7BZ94;  F5H8J2;  I3L398;  I3L312 | Protein disulfide-isomerase | P4HB |
| -0.3066803 | 0.2162376 | 8.137005776 | -3.320481424 | Q9NZM1-6;  Q9NZM1;  F8W8J4;  Q9NZM1-2;  Q9NZM1-3;  C9JCN0;  Q9NZM1-5;  H0YD14;  Q9NZM1-7;  Q9NZM1-8;  Q9NZM1-4;  O75923-15;  O75923-3;  O75923-9;  O75923;  O75923-14;  O75923-6;  O75923-12;  O75923-5;  O75923-11;  O75923-4;  O75923-10;  O75923-2;  O75923-8;  O75923-7;  O75923-13 | Q9NZM1-6;  Q9NZM1;  F8W8J4;  Q9NZM1-2;  Q9NZM1-3;  C9JCN0;  Q9NZM1-5;  H0YD14 | Myoferlin | MYOF |
| -0.5627048 | 0.1988712 | 6.96806246 | -4.162451708 | P07384;  E9PRM1;  E9PIA9;  E9PLX0;  E9PJJ3;  E9PLC9;  E9PMC6;  E9PJA6;  E9PSA6;  E9PQB3;  E9PL37 | P07384 | Calpain-1 catalytic subunit | CAPN1 |
| -0.6647629 | 0.1749178 | 8.256693698 | -14.03055158 | P62258;  P62258-2;  K7EM20;  B4DJF2;  I3L3T1;  K7EIT4;  I3L0W5 | P62258;  P62258-2 | 14-3-3 protein epsilon | YWHAE |
| 0.5563068 | 0.1717192 | 7.903062843 | 3.510146378 | Q5SRN7;  P30455;  P30443;  P04439;  Q5SRN5;  P13746;  P13746-2;  P05534;  P18465;  Q09160;  P30447 | Q5SRN7;  P30455;  P30443;  P04439;  Q5SRN5;  P13746;  P13746-2;  P05534;  P18465;  Q09160;  P30447 | HLA class I histocompatibility antigen, A-36 alpha chain;  HLA class I histocompatibility antigen, A-1 alpha chain;  HLA class I histocompatibility antigen, A-3 alpha chain;  HLA class I histocompatibility antigen, A-11 alpha chain;  HLA class I histocompatibility antigen, A-24 alpha chain;  HLA class I histocompatibility antigen, B-57 alpha chain;HLA class I histocompatibility antigen, A-80 alpha chain;  HLA class I histocompatibility antigen, A-23 alpha chain | HLA-A;  HLA-B |
| -0.6003805 | 0.1183597 | 7.138271112 | -8.180576895 | P19634;  B4DTZ6;  P19634-2;  B1ALD5;  Q9UBY0 | P19634;  B4DTZ6 | Sodium/hydrogen exchanger 1;  Sodium/hydrogen exchanger | SLC9A1 |
| -0.4994834 | 0.08664794 | 8.455423534 | -3.396119975 | P21589;  P21589-2;  Q96B60;  H0Y7R7;  H0Y3X5 | P21589;  P21589-2;  Q96B60 | 5-nucleotidase | NT5E |
| 0.9564285 | 0.08447251 | 8.359987174 | 8.433963573 | Q13308-4;  Q13308;  Q13308-6;  Q13308-2;  Q13308-3;  Q13308-5;  Q86X91;  C9JQR6;  H0Y8F1;  H7C5L0;  F8WDG7;  C9J9E8 | Q13308-4;  Q13308;  Q13308-6;  Q13308-2;  Q13308-3;  Q13308-5 | Inactive tyrosine-protein kinase 7 | PTK7 |
| 0.6789733 | 0.04670055 | 8.375992639 | 8.621075913 | P09619;  E5RJ14;  E5RH16;  E5RII0 | P09619 | Platelet-derived growth factor receptor beta | PDGFRB |
| -0.4270909 | 0.04348443 | 9.629317837 | -4.055601235 | P15144;  H0YLZ8;  H0YMC1 | P15144 | Aminopeptidase N | ANPEP |
| 0.5335633 | -0.01055578 | 8.392274762 | 2.700304568 | P08648;  H0YIV7;  H0YHL9 | P08648 | Integrin alpha-5;  Integrin alpha-5 heavy chain;  Integrin alpha-5 light chain | ITGA5 |
| -0.5766908 | -0.0157534 | 7.15112459 | -5.294846013 | Q9UKU6;  H0YHU0 | Q9UKU6 | Thyrotropin-releasing hormone-degrading ectoenzyme | TRHDE |
| -0.55457 | -0.1054266 | 7.880264663 | -2.569605347 | P26038;  V9GZ54;  E9PNP4 | P26038 | Moesin | MSN |
| 0.9892844 | -0.110106 | 8.187012807 | 8.168732263 | P02786;  G3V0E5;  F5H6B1;  H7C3V5;  F8WBE5 | P02786;  G3V0E5;  F5H6B1 | Transferrin receptor protein 1;  Transferrin receptor protein 1, serum form | TFRC |
| 0.4388251 | -0.1316134 | 7.05579866 | 3.526316179 | O43155 | O43155 | Leucine-rich repeat transmembrane protein FLRT2 | FLRT2 |
| -0.42523 | -0.1644154 | 7.369457065 | -3.213612012 | P00338;  P00338-3;  F5GXY2;  P00338-5;  P00338-2;  F5GYU2;  P00338-4;  F5GXH2;  F5H5J4;  F5H6W8;  F5GZQ4;  F5H8H6;  F5GXC7;  F5GWW2;  F5GXU1 | P00338;  P00338-3;  F5GXY2;  P00338-5;  P00338-2;  F5GYU2;  P00338-4;  F5GXH2 | L-lactate dehydrogenase A chain | LDHA |
| 0.3280323 | -0.1646094 | 7.055722266 | 3.983196135 | Q14974;  Q14974-2;  F5H4R7;  J3KTM9;  J3QRG4;  J3QR48;  J3QKQ5 | Q14974;  Q14974-2;  F5H4R7;  J3KTM9 | Importin subunit beta-1 | KPNB1 |
| 0.02162186 | -0.1848017 | 7.348713647 | 4.411012317 | B7Z6M1;  F8W8D8;  B4DGB4;  P13797;  U3KQI3;  H7C4N2;  Q14651;  B4DI60;  Q5TBN3;  C9JAM8 | B7Z6M1;  F8W8D8;  B4DGB4;  P13797 | Plastin-3 | PLS3 |
| 0.1071523 | -0.1989407 | 8.425469006 | 4.169956718 | P04075;  J3KPS3;  H3BQN4;  P04075-2;  H3BUH7;  H3BR04;  H3BU78;  H3BPS8;  H3BR68;  H3BMQ8 | P04075;  J3KPS3;  H3BQN4;  P04075-2;  H3BUH7;  H3BR04;  H3BU78;  H3BPS8 | Fructose-bisphosphate aldolase A;  Fructose-bisphosphate aldolase | ALDOA |
| 0.1202192 | -0.2422258 | 7.862238747 | 3.677566146 | P22314;  Q5JRR6;  Q5JRS1;  Q5JRS3;  Q5JRS2;  Q5JRR9;  Q5JRS0 | P22314 | Ubiquitin-like modifier-activating enzyme 1 | UBA1 |
| 0.2662768 | -0.259461 | 8.07191881 | 11.18209612 | Q07954;  Q6PJ72;  H0YJI8;  Q86SW0;  Q7Z7K9 | Q07954 | Prolow-density lipoprotein receptor-related protein 1;  Low-density lipoprotein receptor-related protein 1 85 kDa subunit;  Low-density lipoprotein receptor-related protein 1 515 kDa subunit;  Low-density lipoprotein receptor-related protein 1 intracellular domain | LRP1 |
| 0.4471503 | -0.3241694 | 7.203468052 | 4.00383768 | P41250 | P41250 | Glycine--tRNA ligase | GARS |
| 0.5089356 | -0.3293228 | 7.45054162 | 5.038680592 | E5RIP4;  Q15043-2;  Q15043-3;  Q15043;  E5RFZ8;  E5RGA7;  E5RJ40;  E5RJG5 | E5RIP4;  Q15043-2;  Q15043-3;  Q15043;  E5RFZ8;  E5RGA7;  E5RJ40;  E5RJG5 | Zinc transporter ZIP14 | SLC39A14 |
| 0.606916 | -0.3416286 | 7.711765062 | 7.328882975 | P50990;  P50990-2;  P50990-3;  H7C4C8;  H7C2U0 | P50990;  P50990-2;  P50990-3 | T-complex protein 1 subunit theta | CCT8 |
| 0.08637615 | -0.3646449 | 7.574922105 | 2.920172509 | Q9HCU0 | Q9HCU0 | Endosialin | CD248 |
| 0.3490255 | -0.3903963 | 7.376029182 | 18.19770867 | Q13085;  Q13085-4;  Q13085-3;  Q13085-2;  Q59FY4;  K4DID9;  O00763-2;  F8W8T8;  O00763;  K7EII5;  S4R3S7;  K7EK64;  K7EPF1;  D3YTK0;  K7EJ22;  X6RCE4;  A8MYL5;  F6QQI9;  H0YGH5 | Q13085;  Q13085-4;  Q13085-3;  Q13085-2 | Acetyl-CoA carboxylase 1;  Biotin carboxylase | ACACA |
| 0.6629347 | -0.3944108 | 8.361557861 | 13.83940922 | P00533;  E9PFD7;  Q504U8;  P00533-4;  P00533-3;  P00533-2;  C9JYS6;  H3BLT0;  Q15303-4;  Q15303-3;  Q15303-2;  Q15303 | P00533;  E9PFD7;  Q504U8 | Epidermal growth factor receptor | EGFR |
| 0.1522483 | -0.3949608 | 7.993859296 | 2.949855327 | P60842;  J3KT12;  P60842-2;  J3QL43;  J3QS69;  J3QR64;  J3KSZ0;  J3KTB5;  J3QLN6;  J3KTN0;  J3QKZ9;  J3KS25;  J3QL52;  B4E102;  J3KT04;  J3KS93;  E7EMV8;  J3KSN7 | P60842;  J3KT12;  P60842-2;  J3QL43;  J3QS69;  J3QR64;  J3KSZ0;  J3KTB5 | Eukaryotic initiation factor 4A-I | EIF4A1 |
| 0.6243753 | -0.4111571 | 7.722000114 | 3.727359961 | P09651-3;  F8W6I7;  P09651-2;  P09651;  F8VZ49;  Q32P51;  F8VTQ5;  F8VYN5;  H0YH80;  F8W646 | P09651-3;  F8W6I7;  P09651-2;  P09651;  F8VZ49;  Q32P51;  F8VTQ5;  F8VYN5;  H0YH80 | Heterogeneous nuclear ribonucleoprotein A1;  Heterogeneous nuclear ribonucleoprotein A1-like 2 | HNRNPA1;  HNRNPA1L2 |
| 0.6694804 | -0.4336232 | 7.815517979 | 8.859049323 | A8MUD9;  P18124;  C9JZ88;  C9JIJ5 | A8MUD9;  P18124 | 60S ribosomal protein L7 | RPL7 |
| 1.3236 | -0.439109 | 6.59709158 | 9.740350076 | Q16832;  Q5T245;  Q5T244 | Q16832 | Discoidin domain-containing receptor 2 | DDR2 |
| 0.4825385 | -0.4398329 | 8.342560843 | 11.67645753 | P17813-2;  P17813;  F5GX88 | P17813-2;  P17813;  F5GX88 | Endoglin | ENG |
| 0.2670516 | -0.461005 | 7.230474467 | 3.812095826 | P53618;  E9PP73;  E9PP63;  E9PKQ1 | P53618;  E9PP73 | Coatomer subunit beta | COPB1 |
| 0.6802252 | -0.4643452 | 8.251078264 | 7.589381255 | Q9Y490;  Q5TCU6;  H0YMT1 | Q9Y490;  Q5TCU6 | Talin-1 | TLN1 |
| 0.2559237 | -0.4673738 | 7.229528264 | 6.426431085 | Q8WWI5-3;  Q8WWI5-2;  Q8WWI5;  H9KV47 | Q8WWI5-3;  Q8WWI5-2;  Q8WWI5;  H9KV47 | Choline transporter-like protein 1 | SLC44A1 |
| 0.5252671 | -0.4874394 | 7.895510943 | 10.5798975 | P13639 | P13639 | Elongation factor 2 | EEF2 |
| 0.06818932 | -0.5022803 | 8.785116195 | 4.457904216 | Q5JP53;  P07437;  Q5ST81;  E9PBJ4;  I3L2F9;  A6NNZ2;  M0R042;  Q9H4B7 | Q5JP53;  P07437;  Q5ST81 | Tubulin beta chain | TUBB |
| 0.03337007 | -0.5136254 | 8.268554572 | 3.148735304 | P14618-2;  H3BTN5;  Q504U3;  H3BQ34;  H3BUW1;  H3BTJ2;  H3BT25;  H3BU13;  H3BN34;  H3BQZ3;  P30613-2;  P30613 | P14618-2;  H3BTN5;  Q504U3 | Pyruvate kinase PKM;  Pyruvate kinase | PKM;  PKM2 |
| 0.4067857 | -0.5619652 | 6.660286665 | 5.273753973 | Q9UIQ6;  Q9UIQ6-3;  Q9UIQ6-2 | Q9UIQ6;  Q9UIQ6-3;  Q9UIQ6-2 | Leucyl-cystinyl aminopeptidase;  Leucyl-cystinyl aminopeptidase, pregnancy serum form | LNPEP |
| 0.07628538 | -0.5661218 | 8.790299241 | 2.985573075 | P68363;  C9JDS9 | P68363 | Tubulin alpha-1B chain | TUBA1B |
| 0.433929 | -0.5688156 | 6.776831888 | 9.654982769 | P26639;  P26639-2;  E7ERI3;  G3XAN9;  D6R9F8;  D6RCA5;  D6RDJ6 | P26639;  P26639-2;  E7ERI3;  G3XAN9 | Threonine--tRNA ligase, cytoplasmic | TARS |
| 0.4264258 | -0.5689441 | 7.48784512 | 3.444640687 | Q9BQ51;  Q9BQ51-3;  Q9BQ51-2 | Q9BQ51;  Q9BQ51-3;  Q9BQ51-2 | Programmed cell death 1 ligand 2 | PDCD1LG2 |
| 0.5047729 | -0.5904408 | 7.740978162 | 4.313465548 | J3KPE3;  P63244;  H0YAF8;  H0Y8W2;  D6RHH4;  H0YAM7;  D6RFX4;  D6R9Z1;  D6RAC2;  D6R9L0;  D6RFZ9;  D6REE5;  D6RAU2;  E9PD14;  D6RF23;  H0Y8R5;  D6RBD0;  H0Y9P0;  D6RHJ5;  D6RDI0;  D6RGK8;  D6R909 | J3KPE3;  P63244;  H0YAF8;  H0Y8W2;  D6RHH4;  H0YAM7;  D6RFX4;  D6R9Z1;  D6RAC2;  D6R9L0;  D6RFZ9;  D6REE5 | Guanine nucleotide-binding protein subunit beta-2-like 1 | GNB2L1 |
| 0.740651 | -0.5974088 | 7.98562404 | 8.589858118 | Q16555;  Q16555-2;  E5RFU4;  E9PD68;  Q14194;  Q14194-2 | Q16555;  Q16555-2 | Dihydropyrimidinase-related protein 2 | DPYSL2 |
| 0.1028378 | -0.6254985 | 7.242342621 | 4.133167956 | B4DJV2;  O75390;  H0YIC4;  F8VTT8;  F8W4S1;  F8W642;  F8W0J2;  F8VZK9;  F8VP03;  F8VX07;  F8VRP1;  F8VX68;  F8VWQ5;  F8VPF9;  F8VU34;  F8VPA1;  F8VR34;  F8W1S4;  H0YH82 | B4DJV2;  O75390;  H0YIC4 | Citrate synthase;Citrate synthase, mitochondrial | CS |
| 0.04054174 | -0.6334343 | 8.045909467 | 7.666544775 | P68371 | P68371 | Tubulin beta-4B chain | TUBB4B |
| 0.4235244 | -0.6454482 | 7.36763542 | 8.031518914 | Q01813;  Q01813-2;  B1APP8;  H0Y3Y3;  Q5VSR5;  B1APP6;  H0Y757;  V9GYV7;  V9GY25 | Q01813;  Q01813-2 | 6-phosphofructokinase type C | PFKP |
| 0.6507646 | -0.6945081 | 7.530699042 | 10.45569552 | Q9UNN8 | Q9UNN8 | Endothelial protein C receptor | PROCR |
| 0.3482324 | -0.7307753 | 7.67972762 | 20.37109029 | P30041 | P30041 | Peroxiredoxin-6 | PRDX6 |
| 0.4875892 | -0.7504454 | 6.876835347 | 11.17526694 | P23470-2;  P23470 | P23470-2;  P23470 | Receptor-type tyrosine-protein phosphatase gamma | PTPRG |
| 0.6642097 | -0.7527287 | 7.291613017 | 5.028380226 | Q15582;  G8JLA8;  H0Y8L3;  S4R3C6;  H0Y9D7;  H0YAB8;  D6RBX4;  H0YAH8 | Q15582;  G8JLA8;  H0Y8L3 | Transforming growth factor-beta-induced protein ig-h3 | TGFBI |
| 0.08610431 | -0.7629748 | 7.050070264 | 3.529912298 | P12236;  I7HJJ0 | P12236;  I7HJJ0 | ADP/ATP translocase 3 | SLC25A6 |
| 0.5425057 | -0.7708058 | 7.586992157 | 7.967525457 | O00299 | O00299 | Chloride intracellular channel protein 1 | CLIC1 |
| 0.09410089 | -0.7786054 | 8.61381012 | 18.58600268 | P08238;  Q58FF7;  Q5T9W8;  Q58FF6;  Q58FG1 | P08238 | Heat shock protein HSP 90-beta | HSP90AB1 |
| 0.1139672 | -0.7788777 | 8.111598525 | 2.647299672 | P50454;  E9PPV6;  E9PR70;  E9PKH2;  E9PK86;  E9PMI5;  E9PNX1;  E9PJH8;  E9PIG2;  E9PRS3;  E9PQ34;  E9PLA6;  H0YEP8 | P50454;  E9PPV6;  E9PR70;  E9PKH2 | Serpin H1 | SERPINH1 |
| 0.7225292 | -0.8086142 | 7.169233451 | 7.578278132 | P78527;  E7EUY0;  P78527-2;  H0YG84 | P78527;  E7EUY0;  P78527-2 | DNA-dependent protein kinase catalytic subunit | PRKDC |
| 0.009633875 | -0.8526151 | 8.339411687 | 6.952445549 | P68104;  Q5VTE0;  Q05639;  Q5JR01;  A6PW80 | P68104;  Q5VTE0;  Q05639 | Elongation factor 1-alpha 1;  Putative elongation factor 1-alpha-like 3;  Elongation factor 1-alpha 2 | EEF1A1;  EEF1A1P5;  EEF1A2 |
| 0.6031219 | -0.871997 | 7.744379051 | 25.04900951 | O14786;  E9PEP6;  O14786-3;  E7EX60;  O14786-2;  Q5T7F0;  Q5JWQ4;  Q5JWQ6;  H0Y4A0;  Q5JWQ2 | O14786;  E9PEP6;  O14786-3;  E7EX60;  O14786-2;  Q5T7F0;  Q5JWQ4;  Q5JWQ6 | Neuropilin-1 | NRP1 |
| 1.518031 | -0.8916695 | 8.65394537 | 16.34822065 | P08473;  C9JR96;  C9IYX7;  C9J9X7;  C9JDZ3;  Q3KQS6 | P08473 | Neprilysin | MME |
| 0.4483725 | -0.8953682 | 7.686636269 | 4.318938805 | Q08211;  Q08211-2 | Q08211 | ATP-dependent RNA helicase A | DHX9 |
| 0.8336903 | -0.9452013 | 6.757502312 | 10.36198129 | P11586;  F5H2F4;  V9GYY3 | P11586;  F5H2F4 | C-1-tetrahydrofolate synthase, cytoplasmic;  Methylenetetrahydrofolate dehydrogenase;  Methenyltetrahydrofolate cyclohydrolase;  Formyltetrahydrofolate synthetase;  C-1-tetrahydrofolate synthase, cytoplasmic, N-terminally processed | MTHFD1 |
| 0.02772026 | -0.983789 | 7.240224654 | 10.83514694 | E9PCY7;  P31943;  G8JLB6;  D6RIU0;  D6RBM0;  E7EQJ0;  D6RAM1;  E5RGV0;  D6R9T0;  D6RFM3;  D6RIT2;  E7EN40;  D6RDU3;  D6RJ04;  D6RIH9;  H0YB39;  D6RDL0;  F5GZT4;  H0YBG7;  H0YBD7;  E5RJ94;  D6R9D3;  D6RF17;  E5RGH4;  H0YAQ2 | E9PCY7;  P31943;  G8JLB6;  D6RIU0;  D6RBM0;  E7EQJ0;  D6RAM1;  E5RGV0;  D6R9T0;  D6RFM3;  D6RIT2;  E7EN40;  D6RDU3;  D6RJ04;  D6RIH9;  H0YB39 | Heterogeneous nuclear ribonucleoprotein H;  Heterogeneous nuclear ribonucleoprotein H, N-terminally processed | HNRNPH1 |
| 1.17932 | -0.9923528 | 7.536331136 | 29.20368216 | P55287;  H3BUU9;  P55287-2;  H3BQH2 | P55287;  H3BUU9;  P55287-2;  H3BQH2 | Cadherin-11 | CDH11 |
| 1.236646 | -1.016687 | 7.274550385 | 20.45869937 | Q15758;  M0QXM4;  Q15758-3;  Q15758-2;  M0QX44;  M0R144 | Q15758;  M0QXM4;  Q15758-3;  Q15758-2 | Neutral amino acid transporter B(0) | SLC1A5 |
| 0.8900964 | -1.023622 | 8.537579853 | 8.2437182 | P24821;  P24821-3 | P24821;  P24821-3 | Tenascin | TNC |
| 0.3082541 | -1.108425 | 7.175627764 | 11.05602579 | P11413;  P11413-3;  P11413-2;  E7EUI8;  E7EM57;  E9PD92 | P11413;  P11413-3;  P11413-2;  E7EUI8;  E7EM57;  E9PD92 | Glucose-6-phosphate 1-dehydrogenase | G6PD |
| 0.9256737 | -1.129905 | 7.028774527 | 6.463470132 | O15260-2;  Q5T8U5;  O15260;  O15260-3 | O15260-2;  Q5T8U5;  O15260;  O15260-3 | Surfeit locus protein 4 | SURF4 |
| 0.4955928 | -1.143054 | 9.640382045 | 4.742145806 | P02751-3;  P02751;  P02751-17;  P02751-7;  P02751-15;  P02751-5;  F8W7G7;  P02751-14;  P02751-8;  P02751-9;  P02751-10;  P02751-6;  P02751-11;  P02751-13;  P02751-4;  P02751-12;  H0Y7Z1;  P02751-16;  P02751-2;  H0Y4K8 | P02751-3;  P02751;  P02751-17;  P02751-7;  P02751-15;  P02751-5;  F8W7G7;  P02751-14;  P02751-8;  P02751-9;  P02751-10;  P02751-6;  P02751-11;  P02751-13;  P02751-4;  P02751-12;  H0Y7Z1 | Fibronectin;  Anastellin;  Ugl-Y1;  Ugl-Y2;  Ugl-Y3 | FN1 |
| 1.008057 | -1.144584 | 6.928190948 | 9.978322065 | O94813-3;  O94813-2;  O94813;  X6R3P0;  E9PCX4;  H0Y968;  Q5T0V3;  Q5T0V4;  O75093-2;  Q5T0V0;  O75093 | O94813-3;  O94813-2;  O94813;  X6R3P0 | Slit homolog 2 protein;  Slit homolog 2 protein N-product;  Slit homolog 2 protein C-product | SLIT2 |
| 0.4177591 | -1.290321 | 8.063783561 | 31.95225824 | P49327 | P49327 | Fatty acid synthase;  [Acyl-carrier-protein] S-acetyltransferase;  [Acyl-carrier-protein] S-malonyltransferase;  3-oxoacyl-[acyl-carrier-protein] synthase;  3-oxoacyl-[acyl-carrier-protein] reductase;  3-hydroxyacyl-[acyl-carrier-protein] dehydratase;  Enoyl-[acyl-carrier-protein] reductase;  Oleoyl-[acyl-carrier-protein] hydrolase | FASN |
| 0.1541943 | -1.738072 | 7.699777042 | 17.96160289 | P10809;  E7ESH4;  E7EXB4;  B7Z712;  C9JL25;  C9JL19;  C9JCQ4;  C9J0S9 | P10809 | 60 kDa heat shock protein, mitochondrial | HSPD1 |
| 0.9098883 | -1.741541 | 7.318960229 | 11.11732347 | P23219-4;  P23219-3;  P23219-2;  P23219;  P23219-6;  P23219-5;  X6RJD6 | P23219-4;  P23219-3;  P23219-2;  P23219;  P23219-6;  P23219-5 | Prostaglandin G/H synthase 1 | PTGS1 |
| 1.250658 | -1.893089 | 7.618612835 | 19.03813169 | O43175;  Q5SZU1 | O43175;Q5SZU1 | D-3-phosphoglycerate dehydrogenase | PHGDH |
| 1.381006 | -2.717192 | 8.426250932 | 44.01747361 | P12110;  P12110-3;  P12110-2;  C9JH44;  H7C0M5 | P12110;  P12110-3;  P12110-2 | Collagen alpha-2(VI) chain | COL6A2 |
| 1.206643 | -2.719661 | 9.101918834 | 75.87730302 | P12111-2;  P12111;  E7ENL6;  E9PCV6;  P12111-4;  P12111-5;  P12111-3;  C9JNG9;  I3L392 | P12111-2;  P12111;  E7ENL6;  E9PCV6;  P12111-4;  P12111-5 | Collagen alpha-3(VI) chain | COL6A3 |
| 1.430392 | -2.94586 | 8.688215086 | 42.15969595 | P12109 | P12109 | Collagen alpha-1(VI) chain | COL6A1 |
